# SIRT7 antagonizes TGF-β signaling and inhibits breast cancer metastasis

**DOI:** 10.1038/s41467-017-00396-9

**Published:** 2017-08-22

**Authors:** Xiaolong Tang, Lei Shi, Ni Xie, Zuojun Liu, Minxian Qian, Fanbiao Meng, Qingyang Xu, Mingyan Zhou, Xinyue Cao, Wei-Guo Zhu, Baohua Liu

**Affiliations:** 1 Department of Biochemistry and Molecular Biology, Shenzhen University Health Science Center, Shenzhen, 518060 China; 2 Center for Anti-aging and Regenerative Medicine, Shenzhen University Health Science Center, Shenzhen, 518060 China; 3 Shenzhen Second People’s Hospital, First Affiliated Hospital of Shenzhen University, Shenzhen, 518035 China

## Abstract

Distant metastasis is the main cause of breast cancer-related death; however, effective therapeutic strategies targeting metastasis are still scarce. This is largely attributable to the spatiotemporal intratumor heterogeneity during metastasis. Here we show that protein deacetylase SIRT7 is significantly downregulated in breast cancer lung metastases in human and mice, and predicts metastasis-free survival. SIRT7 deficiency promotes breast cancer cell metastasis, while temporal expression of Sirt7 inhibits metastasis in polyomavirus middle T antigen breast cancer model. Mechanistically, SIRT7 deacetylates and promotes SMAD4 degradation mediated by β-TrCP1, and SIRT7 deficiency activates transforming growth factor-β signaling and enhances epithelial-to-mesenchymal transition. Significantly, resveratrol activates SIRT7 deacetylase activity, inhibits breast cancer lung metastases, and increases survival. Our data highlight SIRT7 as a modulator of transforming growth factor-β signaling and suppressor of breast cancer metastasis, meanwhile providing an effective anti-metastatic therapeutic strategy.

## Abstract (web summary)

Metastatic disease is the major reason for breast cancer-related deaths; therefore, a better understanding of this process and its players is needed. Here the authors report the role of SIRT7 in inhibiting SMAD4-mediated breast cancer metastasis providing a possible therapeutic avenue.

## Introduction

Breast cancer is the second common cancer in females^1^. Among various subtypes, the basal-like breast cancers are the most aggressive and usually associated with high rate of recurrence and/or metastasis^2^, which is the main cause of breast cancer-related death^3^. During metastasis, cancer cells undergo progressive genetic/epigenetic and phenotypic changes, which drive dissemination from primary tumors, subsequent intravasation and migration, and final colonization in distant organs. At molecular level, an epithelial-to-mesenchymal transition (EMT) program underlies the dynamic cellular heterogeneity during metastasis^4^, whereby epithelial cells gradually lose polarity and adhesion capacity but acquire mesenchymal traits, i.e., motility and invasiveness^5^. Such spatial and temporal heterogeneity dictates one of the biggest challenges of targeted breast cancer therapy, which mainly rely on the histological and molecular characteristics of primary tumors^6, 7^.

Transforming growth factor-β (TGF-β) is a key regulator of EMT: extracellular TGF-β signal is transduced through the activation of TGF-β receptors (TGFβR) and subsequent phosphorylation of receptor-activated SMADs (R-SMADs), which form heterotrimeric complex with SMAD4 (co-SMAD). The SMAD complex translocates to nucleus and activates expression of EMT transcription factor Snail1/2, Twist1/2, and Zeb1/2, thus facilitating cancer cell migration and invasion^8, 9^. In addition, TGF-β signaling also upregulates a series of genes, such as *ANGPTL4*, *CTGF*, *IL11*, *S100A4*, and *PTHrP*, to further promote metastasis^10, 11^. Intriguingly, at early stage of cancer development, TGF-β signaling rather provides cell growth inhibitory function^12^, owing to increased expression of CDK inhibitor p15^INK4B^, p21^CIP41^, and p57^KIP213^. Moreover, SMAD4, as a central transducer of TGF-β signaling, is highly mutated during clonal evolution and metastases in colorectal and pancreatic cancers^14, 15^. However, such loss-of-function mutations are rare in invasive breast cancers and SMAD4 deficiency rather inhibits metastasis^16^. Mechanism underlying such spatiotemporal regulation of TGF-β signaling merits in-depth investigation.

The incidence and mortality rate of breast cancer increase with age^17^. However the causal links between aging and metastasis are still poorly understood. NAD^+^-dependent deacylase sirtuins are among the most evolutionarily conserved regulators of aging and longevity^18^. In mammals, seven sirtuin members dictate different enzyme activities and distinctive targets^18^. SIRT7 is the only sirtuin that localizes in nucleolus. It deacetylates H3K18ac thus maintaining oncogenic transformation^19^, and desuccinylates H3K122 to regulate chromatin remodeling during DNA repair^20^. SIRT7 also interacts with mTOR and GTF3C1, a component of the Pol III transcription factor TFIIIC2 complex, to regulate protein synthesis^21^, and cooperates with Myc to suppress ribosome biogenesis^22^. Loss of *Sirt7* in mice causes genomic instability and accelerated aging^23^. Here, utilizing various mouse models and human clinical samples, we uncover a unique function of SIRT7 in suppressing EMT and metastasis via TGF-β signaling. SIRT7 deacetylates and promotes the degradation of SMAD4. SIRT7 is downregulated during metastasis, and predicts lung metastasis-free survival of breast cancers. Resveratrol directly activates SIRT7 deacetylase to suppress TGF-β signaling, thus inhibiting breast cancer metastasis. Our data identify SIRT7 as a novel anti-metastatic therapeutic target and resveratrol as a candidate molecule with potential anti-metastatic benefits.

## Results

### SIRT7 is associated with breast cancer lung metastasis

Aging is the biggest risk factor of cancer and breast cancer mortality increases with age. Given distant metastasis is the main cause of breast cancer-related death, we therefore employed web tool PROGgene V2^24^ and GenAge Human Genes list^25^ to screen for aging genes that are correlated with breast cancer lung metastasis (58 cases)^26^. Significantly, 41 out of 305 genes are correlated with lung metastasis-free survival with statistical significance (Supplementary Data 1). About 39 of the 41 genes are also correlated with relapse-free survival (RFS) in independent cohorts of breast cancers (3554 cases) in Kaplan–Meier database^27^. To obtain more in-depth mechanistic data, we specialized DMFS analysis to basal-like and node-positive breast cancers. At last, 9 aging genes were significantly associated with breast cancer prognosis (Supplementary Data 1). We are particularly interested in SIRT7, which belongs to NAD^+^-dependent deacylase and/or ADP-ribosyltransferase sirtuin family. Loss of *Sirt7* accelerates aging in mice, with cardiac hypertrophy, inflammatory response, and kyphosis^23,28^. However, the exact function of SIRT7 in breast cancer progression and metastasis is still lack of experimental evidences.

**Fig. 1:**
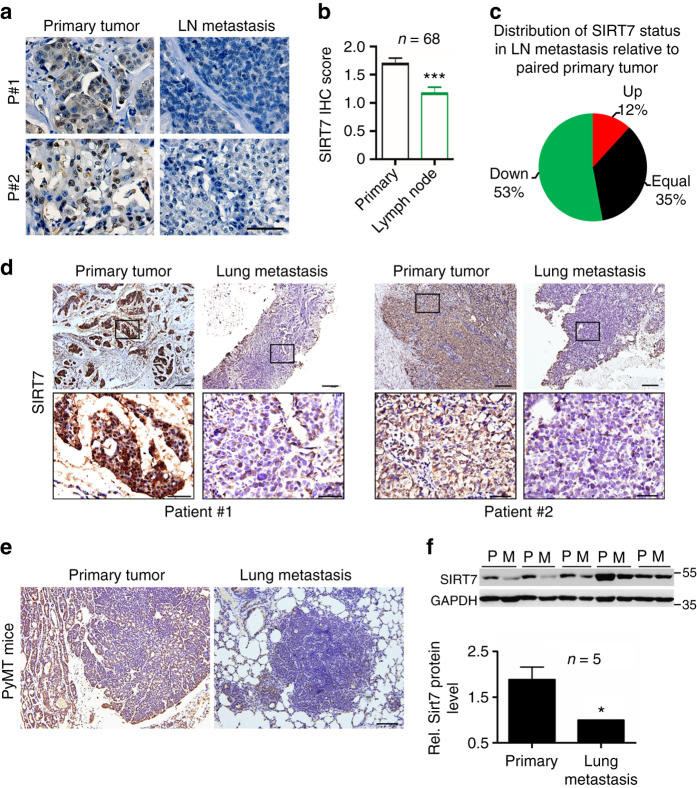
SIRT7 is associated with breast cancer lung metastasis. **a** Representative immunohistochemical (IHC) staining of SIRT7 in primary breast tumors and paired lymph node metastases. *Scale bar*, 50 μm. **b** Average SIRT7 IHC scores in primary breast tumors and lymph node metastases (*n* = 68). **c** Percentage of *SIRT7* expression distribution (up, down, or equal) in lymph node metastasis relative to the paired primary tumor. **d** Representative IHC staining of SIRT7 in primary breast tumors and paired lung metastases. *Scale bar*, 200 μm (*upper*), 50 μm (*lower*). **e** Representative IHC staining of SIRT7 in paired primary tumors and lung metastases in PyMT mice. *Scale bar*, 200 μm. **f** Representative immunoblots showing Sirt7 expression in cells isolated from primary (P) and paired lung metastatic tumors (M) of PyMT mice (*n* = 5). Relative Sirt7 protein levels were quantified by Image J. Data are shown as mean ± S.E.M. *P < 0.05, ***P < 0.001. *P-*values are calculated by Student’s *t*-test

SIRT7 was inversely correlated with poor prognosis in breast cancers (Supplementary Fig. 1a–c). This prompted us to further examine its protein levels in human metastatic tissues. As shown, overall SIRT7 level was significantly downregulated in lymph node metastases compared to paired primary tumors (Fig. 1a, b; Supplementary Fig. 2a); more than 50% node metastases had low levels of SIRT7 relative to the paired primary tumors from the same individuals (Fig. 1c). The overall clinicopathologic parameters were summarized in Supplementary Data 2. We then extended the investigation to lung metastases. Remarkably, SIRT7 was dramatically downregulated in lung metastases in comparison to paired primary tumors in 5 out of 7 patients (Fig. 1d; Supplementary Fig. 2b), suggesting a strong correlation between SIRT7 and human breast cancer lung metastasis.

To further gain in vivo evidences supporting the connection between SIRT7 and breast cancer metastasis, we employed the polyomavirus middle T antigen (PyMT) transgenic mice, a well-investigated breast cancer model with high risk of lung metastasis^29^. PyMT induces breast tumors resembling human luminal-subtype breast cancer at early stage and BLBCs in later stage^30^. All five mice engaged in next study developed palpable breast tumors at age of 7 weeks and obvious lung metastases at age of 16 weeks. A pronounced reduction of Sirt7 was observed in lung metastases compared to the paired primary tumors, as determined by immunohistochemical staining (Fig. 1e), western blotting (Fig. 1f), and real-time PCR (Supplementary Fig. 2c). Together, the data provide in vivo and clinical evidences supporting a negative correlation between SIRT7 and breast cancer lung metastasis.

### SIRT7 prevents breast cancer lung metastasis

**Fig. 2:**
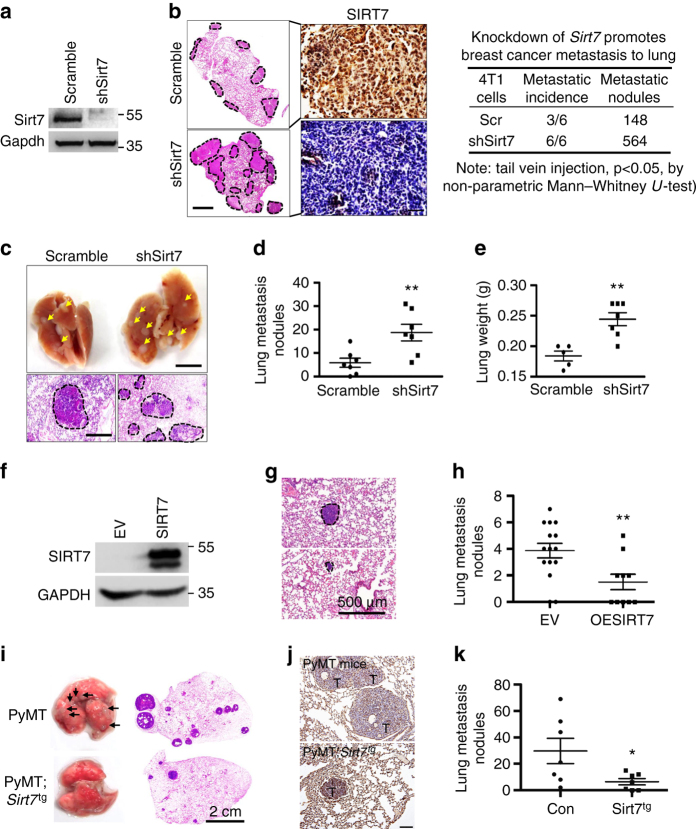
SIRT7 inhibits breast cancer lung metastasis. **a** Immunoblots showing Sirt7 level in 4T1 cells expressing shSirt7. **b** Representative photos of murine lung sections showing metastatic nodules (hematoxylin & eosin staining) (H&E) and Sirt7 expression (*left*). *Dotted lines* indicate the metastatic lesions. *Scale bar*, 1 cm (H&E staining) and 100 μm (IHC). Metastatic incidence and number of nodules were summarized (*right*, *n* = 6 for each group). **c** Representative images of lung (*upper*) and H&E-stained lung sections (*lower*) showing spontaneous metastases generated from 4T1 cells after mammary fat pad injection. *Dotted lines* indicate metastatic lesions. *Scale bar*, 1 cm (*upper*) and 500 μm (*lower*). **d** Scatter plot showing lung metastatic nodules (*n* = 7 for each group). **e** Scatter plot showing the wet weight of lungs in **c**. **f** Immunoblots of SIRT7 in control (empty vector, EV) and SIRT7-overexpressing MDA-MB-231 cells (OESIRT7). **g** Representative H&E-stained lung sections. *Dotted lines* highlight the metastatic lesions. *Scale bar*, 500 μm. **h** Scatter plot showing lung metastatic nodules from animals injected with EV or OESIRT7 cells (*n* = 13 and 10, respectively). **i** Representative images of lung (*left*) and H&E-stained lung sections (*right*) from spontaneous metastases generated in PyMT or PyMT; *Sirt7* ^tg^ mice after doxycycline feeding. *Scale bar*, 2 cm. **j** Representative IHC staining of Sirt7 in lungs of PyMT or PyMT; *Sirt7* ^tg^ mice after doxycycline feeding. *Scale bar*, 100 μm. **k** Scatter plot showing the lung metastatic nodules from control (*n* = 7) or PyMT; *Sirt7* ^tg^ mice (*n* = 7). *P < 0.05, **P < 0.01. *P-*values were obtained by non-parametric Mann–Whitney *U*-test **d**, **h**, **k** or Student’s *t*-test **e**. Data are shown as mean ± S.E.M. and representative of two independent experiments

Consistent with the murine and clinical data, *SIRT7* level is inversely correlated with metastatic capacity of various breast cancer cell lines (Supplementary Fig. 2d, e). To elucidate causal roles of SIRT7 in breast cancer metastasis, we applied highly metastatic murine 4T1 and human MDA-MB-231 cells. About 1 × 10^5^ 4T1 cells expressing either shSirt7 (4T1-shSirt7) or Scramble shRNA (4T1-Scr) were intravenously (i.v.) injected into Balb/c mice. About 4 weeks after inoculation, all mice developed lung metastases by 4T1-shSirt7 cells injection, whereas only half developed by 4T1-Scr cells. An average of 94 metastatic nodules per mouse was observed in 4T1-shSirt7 group, while only 48 were observed in control group (Fig. 2a–b). To closely mimic the in vivo metastatic process, 5 × 10^5^ 4T1 cells were inoculated in inguinal mammary fat pad (i.m.f.p.). After 4 weeks, 4T1-shSirt7 cells developed lung metastases with average 18.7 ± 3.5 nodules per mouse, whereas only 5.9 ± 1.9 nodules were found in 4T1-Scr cells (Fig. 2c, d). Consistently, the wet lung weight of 4T1-shSirt7 cells-beard mice was much heavier than 4T1-Scr cells (Fig. 2e), suggesting more severe metastasis occurred. Of note, knocking down *SIRT7* in 4T1 cells had little effect on cell growth in vitro (Supplementary Fig. 3a, b), but it promoted in vivo primary tumor growth 21 days after inoculation, a later stage of tumor with nutrient limitation (Supplementary Fig. 3d–f).

To further determine the inhibitory role of SIRT7 in lung metastasis, 1 × 10^6^ MDA-MB-231 cells expressing SIRT7 (OESIRT7) or empty vector (EV) were i.v. injected. After 35 days, an average of 4.0 ± 2.0 lung metastatic modules was observed in EV-inoculated mice, while only 1.5 ± 1.8 metastatic modules were found in OESIRT7 (Fig. 2f–h). Similar to 4T1, overexpression of SIRT7 did not affect MDA-MB-231 cell growth. To further confirm the in vivo causal links between Sirt7 and lung metastasis, we generated an inducible *Sirt7* transgenic line (*Sirt7* ^tg^, Supplementary Fig. 4a, b), and bred them to get PyMT; *Sirt7* ^tg^ compound mice. The expression of ectopic *Sirt7* was successfully turned on in various tissues by doxycycline (Dox) feeding (Supplementary Fig. 4c). Then the PyMT, *Sirt7* ^tg^ mice were fed with Dox at 4 weeks of age, when they just developed tumors in hyperplastic stage^31^, and killed for lung metastatic analyses at 16 weeks of age. The induced expression of Sirt7 was observed in primary tumors and isolated tumor cells (Supplementary Fig. 4d). The dox-fed timing was to exclude possible artificial effects resulted from potential mammary developmental defects due to ectopic Sirt7. While the tumor growth and palpable tumor latency were merely affected (Supplementary Fig. 4e), induced expression of Sirt7 remarkably inhibited lung metastasis (Fig. 2i–k; Supplementary Fig. 4f). Collectively, these findings support an inhibitory role of SIRT7 in breast cancer lung metastasis.

### SIRT7 inhibits TGF-β signaling

**Fig. 3:**
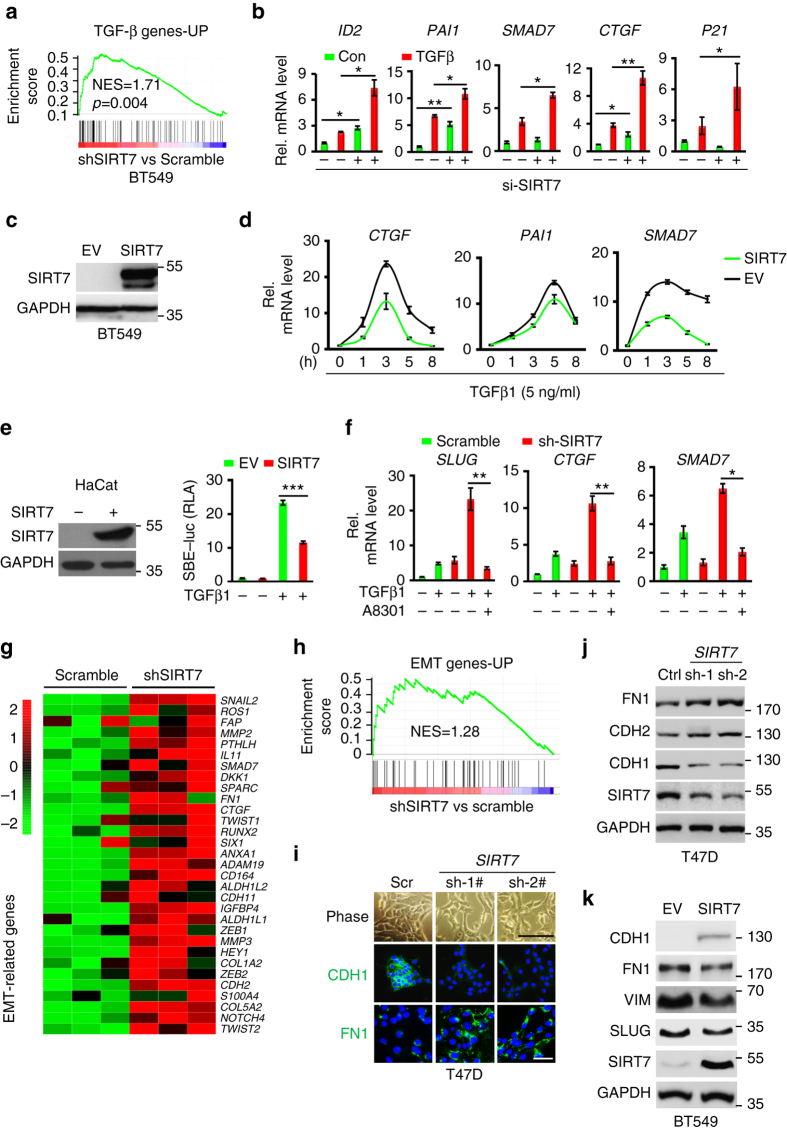
Loss of *SIRT7* activates TGF-β signaling and promotes EMT. **a** Gene set enrichment analysis (GSEA) showing the enrichment of TGF-β signaling gene signatures^69^ in *SIRT7* KD BT549 cells (shSIRT7) compared with Scramble control. **b** Quantitative RT-PCR analysis of TGF-β downstream gene *ID2*, *PAI1*, *SMAD7*, *CTGF*, and *P21* in *SIRT7* KD BT549 cells in the presence or absence of TGF-β1 (5 ng/ml, 2 h). **c** Representative immunoblots showing ectopic SIRT7 expression in BT549 cells. **d** Quantitative RT-PCR analysis of TGF-β signaling downstream gene *CTGF*, *PAI1*, and *SMAD7* at indicated time points in BT549 cells expressing ectopic SIRT7. **e** Relative luciferase activity (RLA) driven by SBE4-luc in control and SIRT7-overexpressing HaCaT cells. **f** Quantitative RT-PCR analysis of TGF-β downstream gene *SLUG*, *SMAD7*, *CTGF* levels in cells treated with TGFβ1 (5 ng/ml) or TGFβR1 inhibitor A8301 (5 µM). **g** Heatmap representation of RNA-Seq data showing expression levels of EMT-related genes^69^ in Scramble and *SIRT7* KD BT549 cells. **h** GSEA showing the enrichment of EMT-related gene signatures^70^ in *SIRT7* KD cells. **i** Phase contrast and immunofluorescence images showing epithelial marker CDH1 (E-cadherin) and mesenchymal marker Fibronectin 1 (*FN1*) in Scramble and *SIRT7* KD T47D cells. *Scale bar*, 100 µm (*bright field*) and 50 µm (IF). **j** Immunoblots of SIRT7 and various EMT markers in T47D cells treated with Scramble (Ctrl) or SIRT7 shRNAs. **k** Immunoblots of SIRT7 and various EMT markers in BT549 cells expressing SIRT7 or empty vector control (*EV*). *P < 0.05, **P < 0.01, *** *P* < 0.001. *P*-values are calculated by Student’s *t*-test. Data are shown as mean ± S.E.M. *NES*: normalized enrichment score

Based on *SIRT7* levels and an online omics tool^32^, we performed KEGG analysis in a TCGA RNA-seq data set obtained from 1097 individuals diagnosed of invasive breast carcinoma. The overall distribution of *SIRT7* level was shown (Supplementary Fig. 5a). A positive correlation between *SIRT7* and overall survival (Supplementary Fig. 5b), and an enrichment of pathway of focal adhesion (*P* = 0.00778), ECM–receptor interaction (*P* = 0.01223), and TGF-β signaling (*P* = 0.03506) among genes that were negatively correlated with *SIRT7*, were observed (Supplementary Fig. 5c, d). Consistently, a negative correlation between *SIRT7* and TGF-β pathways was found in *SIRT7* KD BT549 cells, a triple negative and metastatic site-derived breast cancer cell line, as determined by gene set enrichment analysis (GSEA)^33^ (Fig. 3a). The enhanced TGF-β signaling was confirmed by real-time PCR analysis of a series of genes in the presence or absence of TGF-β1 (Fig. 3b; Supplementary Fig. 6a). Of note, *CTGF*, *MMP1*, and *PTGS2* are among genes that drive breast cancer metastasis to bone^34^ (Supplementary Data 3). On the other front, ectopic SIRT7 inhibited TGF-β-regulated gene expression (Fig. 3c, d). It was confirmed by a luciferase reporter (SBE-Luc) driven by a TGF-β responsive promoter (Fig. 3e). Similarly, knocking down *SIRT7* activated TGF-β signaling in 4T1 and MDA-MB-231 cells, while overexpression of SIRT7 inhibited it (Supplementary Fig. 6b–d). In addition, the enhanced TGF-β signaling was blocked by Alk5 inhibitor A8301 and SB431542 (Fig. 3f; Supplementary Fig. 6e), suggesting a suppressive role of SIRT7 in TGF-β signaling.

**Fig. 4:**
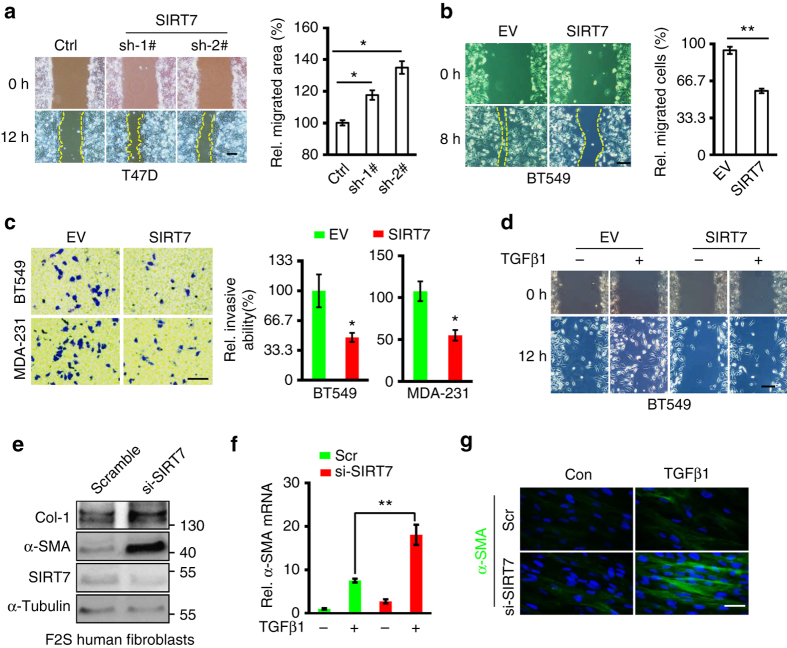
SIRT7 inhibits breast cancer motility. **a**, **b** Wound healing assay of SIRT7 KD T47D cells **a** and SIRT7-overexpressing BT549 cells **b**. *Scale bar*, 100 µm. **c** Representative images of Transwell invasion assay for SIRT7-overexpressing breast cancer BT549 and MDA-MB-231 cells. Cell number was counted in six randomly captured pictures. Invasive ability was normalized to control. *Scale bar*, 100 µm. **d** Wound healing assay in BT549 cells expressing ectopic SIRT7 in the presence or absence of TGF-β1 (5 ng/ml). *Scale bar*, 100 µm. **e** Immunoblots showing Col1a1 and α-SMA in F2S human fibroblast cells treated with Scramble or si-SIRT7. **f**, **g** Quantitative RT-PCR and immunofluorescence staining of α-SMA in the Scramble (Scr) or si-SIRT7-treated F2S cells in the presence or absence of TGF-β1 (5 ng/ml). *Scale bar*, 50 µm. Data are shown as mean ± S.E.M. *P < 0.05, **P < 0.01. *P*-values are calculated by Student’s *t*-test

TGF-β is a key regulator of EMT, which is critical for breast cancer heterogeneity and progression^35^. Indeed, an enrichment of genes that mediate EMT in *SIRT7* KD BT549 cells was observed (Fig. 3g, h). *SIRT7* KD increased mesenchymal traits in T47D and 4T1 breast cancer cells (Fig. 3i, j; Supplementary Fig. 6f, g). On the other side, ectopic SIRT7 inhibited mesenchymal markers in BT549 cells (Fig. 3k; Supplementary Fig. 6h). EMT enables cells migratory and invasive capacity. In line with this notion, knocking down *SIRT7* promoted but ectopic SIRT7 inhibited cell migration in a wound healing assay (Fig. 4a, b). The suppressive role of SIRT7 in cell invasion was confirmed by Transwell assay in BT549 and MDA-MB-231 cells (Fig. 4c). Overexpression of SIRT7 abolished the migration-promoting effects of TGF-β1 (Fig. 4d). SIRT7 knockdown promoted TGF-β-mediated EMT transition and was blocked by Alk5 inhibitor A8301 in T47D cells (Supplementary Fig. 6i, j), further implicating that SIRT7-regulated breast cancer invasive and migratory ability was TGF-β-dependent. The EMT program also promotes skin fibrosis. Consistently, knocking down *SIRT7* in human skin fibroblast cells enhanced expression of *Col1a1* and *α-SMA*, two typical marker genes of fibrosis (Fig. 4e–g). Together, the data implicates that SIRT7 antagonizes TGF-β signaling-regulated EMT and breast cancer invasive ability.

### SIRT7 deacetylates SMAD4

**Fig. 5:**
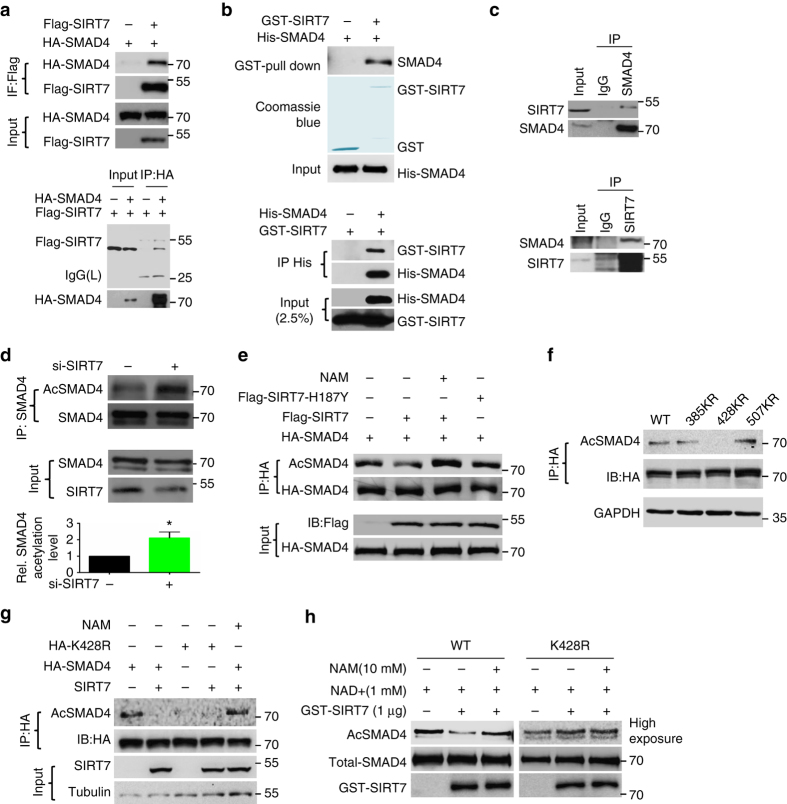
SIRT7 deacetylates SMAD4. **a** Immunoblots showing HA-SMAD4 in the anti FLAG-SIRT7 immunoprecipitates (*upper*) and FLAG-SIRT7 in the anti HA-SMAD4 immunoprecipitates (*lower*). **b** GST pulldown with purified GST-SIRT7 (*upper*) and Ni-NTA pulldown with 6× His-SMAD4 (*lower*) followed by immunoblotting with anti SMAD4 and SIRT7 antibodies. **c** Co-immunoprecipitation of endogenous SMAD4 with anti SIRT7 antibodies (*upper*) and endogenous SIRT7 with anti SMAD4 antibodies (*lower*) in BT549 cells. **d** Acetylation level of SMAD4 in BT549 cells treated with Scramble or si-SIRT7, probed with pan anti acetyl lysine antibodies. *Lower* is quantitative data showing increased level of SMAD4 acetylation in cells with *SIRT7* KD. **e** Immunoblots showing the acetylation levels of SMAD4 in the presence of wild-type, enzyme-dead SIRT7 (H187Y), and/or NAM (10 mM). **f** Immunoblots showing acetylation levels of SMAD4 WT and KR mutants (K385R, K428R, and K507R). These proteins were immunoprecipitated with anti HA affinity resin and probed with pan anti acetyl K antibodies. **g** Immunoblots showing the acetylation levels of SMAD4 WT and K428R mutant in the presence of SIRT7 or NAM. **h** SIRT7-mediated in vitro deacetylation of SMAD4. Bead-bound SMAD4 or K428R mutant was included. *P < 0.05, *P*-value is calculated by Student’s *t*-test. Data are shown as mean ± S.E.M

In canonical TGF-β signaling, activated TGFβR1 phosphorylates SMAD2/3, which then forms heterotrimeric complex with SMAD4, moves to the nucleus, and activate gene expression^36^. However, no phosphorylation changes of SMAD2/3 were observed in cells transfected with shSIRT7 or ectopic *SIRT7* in comparison with mock-treated controls (Supplementary Fig. 7a, b). We then asked whether SIRT7 deacylase activity regulates SMADs. To test this possibility, we first performed co-immunoprecipitation in HEK293 cells expressing ectopic FLAG-SIRT7 and HA-SMADs. As shown, SIRT7 was detected in the anti HA-SMAD4 immunoprecipitates but was completely absent in the HA-SMAD1 and HA-SMAD2/3 immunoprecipitates (Supplementary Fig. 7c; Fig. 5a). Reciprocally, SMAD4 was present in anti FLAG-SIRT7 immunoprecipitates (Fig. 5a). Further, His-tagged SMAD4 was pulled down by GST-SIRT7 and vice versa, indicating physical interaction between SIRT7 and SMAD4 (Fig. 5b). The interaction between endogenous SIRT7 and SMAD4 was also observed (Fig. 5c). In canonical TGF-β pathway, SMAD4 is the only common-mediator SMAD (co-SMAD), consisting of one N-terminal MH1 domain, one C-terminal MH2 domain, and one linker region in between. The MH2 domain mediates the heterotrimeric complex formation^36^. Notably, SIRT7 interacted with both full-length SMAD4 and MH2 domain but not linker region and MH1 domain (Supplementary Fig. 7d). Deleting neither N-terminus (ΔN) nor C-terminus (ΔC) of SIRT7 affected its interaction with SMAD4, and the catalytic domain was enough to pull down SMAD4 (Supplementary Fig. 7e). The interaction is likely SIRT7-specific, as no obvious interaction between SIRT1 and SMAD4 was found (Supplementary Fig. 7f).

The physical interaction between SMAD4 and SIRT7 suggests potential deacetylation regulation. Indeed, the acetyl lysine (K) levels of SMAD4 were substantially enhanced in cell treated with si-*SIRT7*, as determined by anti pan acetyl K antibodies (Fig. 5d). To confirm this observation, wild-type or enzyme-dead SIRT7 (SIRT7-H187Y) was co-transfected with HA-SMAD4 into HEK293 cells and acetylation level of SMAD4 was detected in anti HA immunoprecipitates. As expected, acetyl K levels of HA-SMAD4 was inhibited by SIRT7 (Fig. 5e). The reduction of SMAD4 acetylation was abolished when SIRT7 was replaced by SIRT7-H187Y or inhibited by nicotinamide (NAM) (Fig. 5e). Considering the interaction of MH2 domain and SIRT7, residue K385, K428, and K507 located in this region are likely targeted for SIRT7 deacetylation, as predicted^37, 38^. To test it, K385R-, K428R-, and K507R-mutated HA-SMAD4 were transiently transfected into HEK293 cells. Anti HA immunoprecipitation and western blotting were conducted to determine the acetylation levels of various SMAD4 mutants. As shown, while the acetylation of K428R was almost abolished, that of 385KR and 507KR remained unaffected (Fig. 5f). Moreover, SIRT7-mediated deacetylation was abrogated in K428R mutant (Fig. 5g). This is confirmed in test tube containing recombinant GST-SIRT7 and M2 beads-purified WT or K428R-mutated HA-SMAD4 proteins. While the acetylation of WT SMAD4 was significantly decreased in the presence of NAD^+^ and GST-SIRT7, and inhibited by NAM, the acetylation of K428R remained unchanged (Fig. 5h). These data implicate SMAD4 K428 is subjected for acetylation, and the reverse deacetylation is mediated by SIRT7.

### SIRT7-mediated deacetylation destabilizes SMAD4

The MH2 domain mediates SMAD complex formation. We found the binding capacity of SMAD4 to SMAD2 was deceased in cells expressing ectopic SIRT7 (Supplementary Fig. 8a). The K428R mutation inhibited but K428Q promoted the binding capacity of SMAD4 to SMAD2 (Supplementary Fig. 8a). K428R mutant inhibited nuclear importation but K428Q enhanced nuclear retention of SMAD4, as determined by fluorescence microscopy in cells transfected with GFP-SMADs (Supplementary Fig. 8b). Moreover, downregulation of SIRT7 increased the nuclear retention of SMAD complex, as determined by western blotting in subcellular fractions of cells pre-treated with TGFβ1 and/or TGFβR1 inhibitor A8301 (Supplementary Fig. 8c), suggesting that SIRT7-mediated deacetylation modulates the nuclear-cytoplasmic shuttling of SMAD complex.

**Fig. 6:**
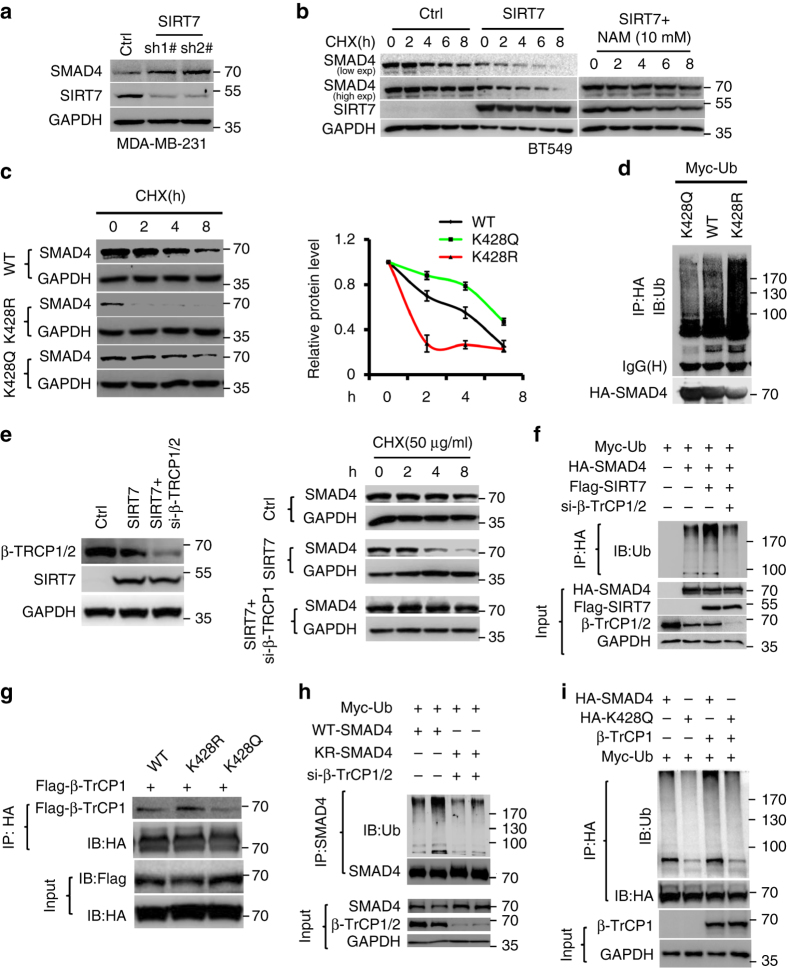
SIRT7 promotes β-TrCP1-mediated SMAD4 degradation. **a** Immunoblots showing SMAD4 protein levels in Scramble (Ctrl) or shSIRT7-treated MDA-MB-231 cells. **b** Immunoblots showing SMAD4 levels in the presence of CHX, with or without ectopic SIRT7 or NAM. **c** Wild-type, K428R, and K428Q mutants of SMAD4 levels in the presence of CHX (50 μg/ml). Quantification was performed by Image J; data were presented as mean ± S.D. **d** Ubiquitination levels of SMAD4 WT and mutants. **e** Immunoblots showing SMAD4 level in cells expressing ectopic SIRT7, treated with control or β-TrCP1 siRNA. **f** Immunoblots showing polyubiquitination of SMAD4 in the presence or absence of ectopic SIRT7 or β-TrCP1 siRNA. **g** Immunoprecipitation showing the binding capacity between Flag-β-TrCP1 and various HA-SMAD4 mutants. **h**, **i** Ubiquitination levels of HA-SMAD4 K428R **h** and K428Q **i** in cells treated with control or β-TrCP1 siRNAs or ectopic β-TrCP1 as indicated

Ubiquitination plays critical roles in nuclear-cytoplasmic shuttling and protein stability of SMAD complex^39, 40^. We then asked whether SIRT7-mediated K428 deacetylation and nuclear-cytoplasmic shuttling destabilizes SMAD4. Compared with mock-treated cells, SMAD4 protein level was significantly increased in various breast cancer cells with *SIRT7* KD (Fig. 6a; Supplementary Fig. 9a). The mRNA level of *SMAD4* remained unaffected (Supplementary Fig. 9b). Consistently, SMAD4 protein level was significantly downregulated when SIRT7 was overexpressed, whereas the mRNA was unchanged (Supplementary Fig. 9c). When protein synthesis was inhibited by cycloheximide (CHX), ectopic SIRT7 promoted the degradation of SMAD4 (Fig. 6b; Supplementary Fig. 9d), and *SIRT7* KD inhibited degradation of SMAD4 (Supplementary Fig. 9e). Moreover, the K428R, mimicking hypo-acetylated SMAD4, accelerated the degradation, whereas the K428Q, resembling hyper-acetylated SMAD4, inhibited the degradation (Fig. 6c). SMAD4 degradation is likely mediated by proteasome, as it was inhibited by MG132 (Supplementary Fig. 9f). Further experiments revealed that deletion of SIRT7 via CRISPR/Cas9 inhibited HA-SMAD4 ubiquitination, while overexpression of SIRT7 increased ubiquitination (Supplementary Fig. 10a, b). The ubiquitination of endogenous SMAD4 is decreased upon *SIRT7* KD (Supplementary Fig. 10c), and K428R mutation promoted SMAD4 ubiquitination (Fig. 6d).

At least four E3 ligases, i.e., SMURF1, SKP2, STUB1, and β-TrCP1, have been shown able to regulate ubiquitination and degradation of SMAD4^41^. SMAD4 level was decreased in cells expressing ectopic STUB1 and β-TrCP1 but not in those expressing SMURF1 and SKP2 (Supplementary Fig. 10d). The accelerated SMAD4 degradation induced by ectopic β-TrCP1 was abolished by NAM treatment, but that induced by STUB1 was hardly affected (Supplementary Fig. 10e, f), suggesting that β-TrCP1 is responsible for SIRT7-mediated SMAD4 degradation. This is supported by the interacting complex of SIRT7, SMAD4, and β-TrCP1 (Supplementary Fig. 10g). Consistently, knocking down β-TrCP1 abolished the increased ubiquitination and accelerated degradation of SMAD4 caused by ectopic SIRT7 (Fig. 6e, f). Moreover, the K428R mutant showed increased binding capacity to β-TrCP1 (Fig. 6g), and elevated ubiquitination, which was restored by β-TrCP1 KD (Fig. 6h). In contrast, K428Q inhibited the ubiquitination of SMAD4 with or without ectopic β-TrCP1 (Fig. 6i). Together, these data indicate that β-TrCP1 is responsible for K428 deacetylation-mediated SMAD4 degradation under the control of SIRT7.

### SMAD4 underlies SIRT7-mediated breast cancer lung metastasis

**Fig. 7:**
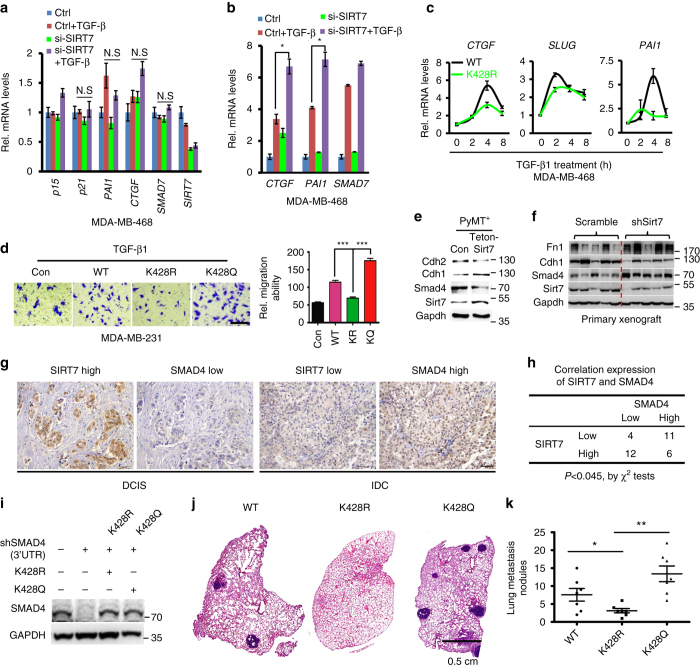
SMAD4 underlies SIRT7-mediated TGF-β signaling. **a** Real-time RT-PCR analysis of TGF-β downstream genes in *SMAD4*-deficient MDA-MB-468 cells treated with control or *SIRT7* siRNA in the presence or absence of TGF-β1 (5 ng/ml, 4 h). **b** Real-time RT-PCR analysis of TGF-β downstream genes in *SMAD4*-restored MDA-MB-468 cells treated with control or *SIRT7* siRNA in the presence or absence of TGF-β1 (5 ng/ml, 4 h). **c** Quantitative RT-PCR analysis of TGF-β signaling genes. MDA-MB-468 cells were reconstituted with WT or K428R SMAD4 and treated with TGF-β1 (5 ng/ml) as indicated. **d** Transwell invasion assay of MDA-MB-231 cells ectopically expressing WT, K428R, or K428Q SMAD4 in the presence of TGF-β1 (5 ng/ml). Endogenous SMAD4 was stably knocked down by shRNA-targeted 3’UTR. Relative invasive ability was normalized to empty vector. **e** Immunoblots showing EMT markers, and negative correlation between Smad4 and Sirt7 levels in primary tumors obtained from PyMT; *Sirt7* ^tg^ and control mice. **f** Immunoblots showing EMT markers, and negative correlation between Smad4 and Sirt7 levels in tumors from mice of mammary fat pad burden with control or *Sirt7* KD 4T1 cells. **g** Representative images of a human breast cancer tissue array showing SIRT7 and SMAD4 protein levels in ductal carcinoma in situ (DCIS, SIRT7 high, and SMAD4 low) and invasive ductal carcinoma (IDC, SIRT7 low, and SMAD4 high). *Scale bar*, 50 µm. **h** Overall correlated expression of SIRT7 and SMAD4 in a human breast cancer tissue array of **g**. **i** Representative immunoblots showing expression of SMAD4s. **j** H&E-stained lung section images showing metastatic nodules. **k** Scatter plot showing lung metastatic nodules in xenograft models (*n* = 7 for each group). *P < 0.05, **P < 0.01, ***P < 0.001. *P-*values are calculated by non-parametric Mann–Whitney *U*-test **k**, Student’s *t*-test **a**, **b**, **d** or *χ* ^2^-test **h**. Data are shown as mean ± S.E.M. N.S., no significance.

To determine the contributing role of SMAD4 in SIRT7-regulated TGF-β signaling, we employed MDA-MB-468 cells, a breast cancer cell line lacking *SMAD4*. As shown, the expression of TGF-β responsive genes like *p15*, *p21*, *PAI1*, *CTGF*, and *SMAD7* was not affected by *SIRT7* KD (Fig. 7a), which was restored by the reconstitution of ectopic SMAD4 (Fig. 7b; Supplementary Fig. 11a). Contrastingly, K428R mutant SMAD4 failed to restore TGF-β signaling in MDA-MB-468 cells (Fig. 7c; Supplementary Fig. 11b). Additionally, while WT and K428Q promoted cell migration in MDA-MB-231 cells, K428R lost the ability (Fig. 7d). These data suggest a SIRT7-SMAD4 axis in regulating TGF-β signaling.

Supporting a SIRT7-SMAD4 axis in the regulation of breast cancer metastasis, negative correlation between Sirt7 and Smad4 protein levels was repeatedly observed in breast tumors isolated from PyMT; *Sirt7* ^tg^ mice and 4T1 xenograft models (Fig. 7e, f). Further, protein levels of SIRT7 and SMAD4 were examined in a human breast cancer tissue array by immunohistochemistry microscopy. The overall clinicopathologic parameters were summarized in Supplementary Data 4. SIRT7 protein was predominantly stained in nucleus and progressively declined during breast cancer progression (Fig. 7g). An inverse significant correlation between SIRT7 and SMAD4 expression was found (Fig. 7h). To validate the potential causal role of SMAD in vivo, we replaced the endogenous gene with K428R and K428Q mutants (see Methods section) and examined lung metastasis in xenograft models. As shown, the replacement of SMAD4 with K428R in MDA-MB-231 cells significantly inhibited lung metastasis, whereas K428Q promoted lung metastasis (Fig. 7i–k). Collectively, these data implicate an essential role of SIRT7-SMAD axis in breast cancer lung metastasis.

### Resveratrol activates SIRT7 and inhibits lung metastasis

**Fig. 8:**
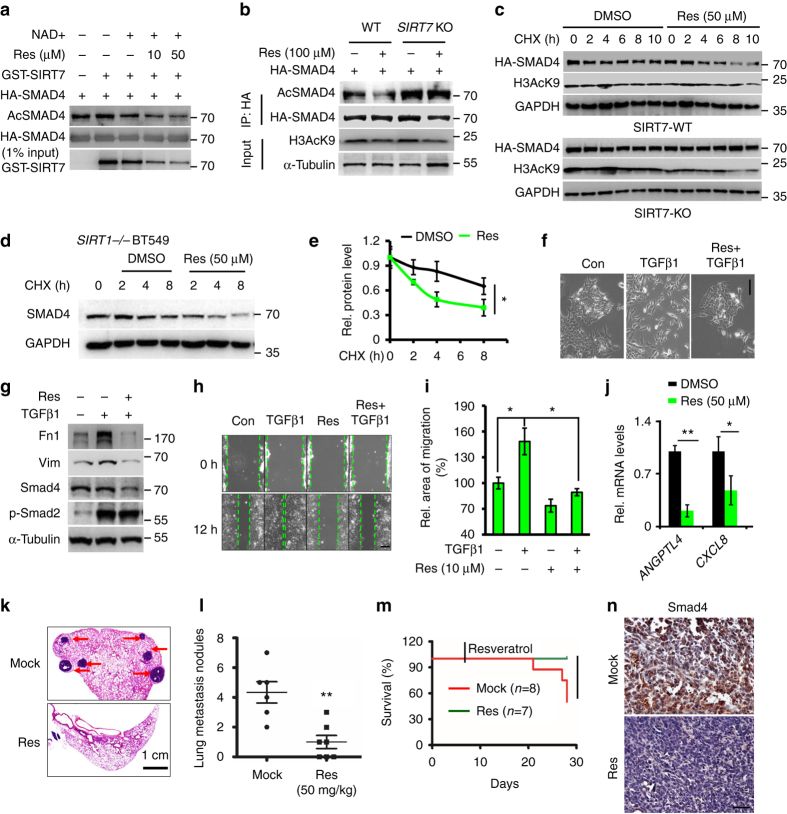
Resveratrol activates SIRT7 and inhibits metastasis. **a** Immunoblots showing SMAD4 acetylation level in an in vitro SIRT7 deacetylation assay: 2 mM NAD^+^, 1 µg GST-SIRT7, bead-bound HA-SMAD4, and Resveratrol (Res) were included as indicated. **b** Immunoblots showing SMAD4 acetylation level in wild-type or *SIRT7* KO HEK293T cells treated with resveratrol. Note the SIRT7-dependent effect of SMAD4 deacetylation and SIRT7-independent effect of H3K9 deacetylation. **c** Resveratrol treatment accelerated the protein degradation of HA-SMAD4, and that was abolished in *SIRT7* KO cells. **d** Representative immunoblots showing accelerated SMAD4 degradation upon resveratrol treatment in *SIRT1* KO BT549 cells. **e** Quantification of **d** by Image J; **f** Phase contrast images of 4T1 cells showing epithelial or mesenchymal features. Noted that Resveratrol inhibited EMT. *Scale bar*, 100 µm. **g** Immunoblots showing decreased Smad4, Fn1, and Vim upon resveratrol treatment. Noted that p-Smad2 levels were not affected. **h** Wound healing assay for 4T1 cells treated with TGF-β1 (5 ng/ml) and/or resveratrol. *Scale bar*, 200 µm. **i** Quantification of migration ability in **h**, represented as relative migration area calculated by Image J. **j** Quantitative RT-PCR analysis of *ANGPTL4* and *CXCL8* mRNA levels in breast cancer cells treated with resveratrol or vehicle. **k** Representative images of H&E staining lung metastatic nodules in Balb/c mice injected with 4T1 cells and treated with resveratrol on day 7 (50 mg/kg). *Scale bar*, 1 cm. **l** Scatter blot showing lung metastatic nodules. **m** Kaplan–Meier survival of mice in **k**. *P < 0.05, calculated by log rank test. **n** Representative IHC staining of Smad4 in lung metastases from 4T1 mice **k**. *Scale bar*, 100 μm. *P < 0.05, **P < 0.01. *P-*values are calculated by one-way analysis of variance (ANOVA) **e**, non-parametric Mann–Whitney *U*-test **l**, Student’s *t*-test **i**, **j** or log rank test **m**. Data are shown as mean ± S.E.M

The above cellular and in vivo evidences suggest SIRT7 inhibits breast cancer lung metastasis. Interestingly, we found, in the presence of GST-SIRT7, resveratrol, a SIRT1 activator, promoted HA-SMAD4 deacetylation in a dose-dependent manner (Fig. 8a). In HEK293 cells treated with resveratrol, the acetylation level of ectopic HA-SMAD4 was decreased, which was abrogated in *SIRT7* KO cells (Fig. 8b). As control, the level of H3AcK9, a known deacetylation target of SIRT1, was decreased in the presence of resveratrol regardless the SIRT7 status. Further, resveratrol treatment accelerated HA-SMAD4 degradation in unmodified control cells but not in *SIRT7* KO cells (Fig. 8c). Consistently, resveratrol treatment promoted the degradation of endogenous SMAD4 in a SIRT1-independent manner (Fig. 8d, e). In addition, we found resveratrol treatment inhibited TGF-β1-induced mesenchymal morphology and gene expression, downregulated expression of SMAD4, and suppressed cell migration in wound healing assay (Fig. 8f–i). More importantly, TGF-β responsive gene *ANGPTL4* and *CXCL8* that are required for breast cancer lung metastasis and drug resistance were significantly inhibited by resveratrol (Fig. 8j).

We then examined whether resveratrol could prevent or inhibit lung metastasis of breast cancer. To this end, Balb/c mice injected with 4T1 cells were employed. About 7 days after inoculation (5 × 10^5^ cells, i.m.f.p.), mice were randomly divided into two groups and were treated with either resveratrol (50 mg/kg, i.p.) or vehicle every other day for another 23 days and then killed for lung metastatic analysis. As shown, 4.33 ± 1.75 metastatic modules were observed in vehicle group, in contrast, only 1.00 ± 1.16 metastatic modules were found in resveratrol group (Fig. 8k, l). Notably, the overall survival at 30 days was 100% in resveratrol-treated group but only 50% in mock-treated mice (Fig. 8m). Consistently, the protein level of Smad4 was largely reduced in resveratrol-treated lung metastases (Fig. 8n). Together, the data suggest SIRT7 against SMAD4 stability as a potential therapeutic target for lung metastasis and beneficial effects of resveratrol in breast cancer treatment.

## Discussion

Effective therapeutic strategies targeting breast cancer metastases are still scarce. The spatiotemporal intratumor cellular heterogeneity dictates the biggest challenge. TGF-β signaling is a well-investigated contributor of breast cancer cellular heterogeneity and metastasis. However, regulators involved in TGF-β signaling during tumor progression remain less understood. Here we found SIRT7 modulates EMT via TGF-β signaling, which is downregulated during metastasis, and predicts lung metastasis-free survival. While *SIRT7* deficiency promotes lung metastasis of breast cancers, temporally induced ectopic expression of *SIRT7* inhibits lung metastasis. Significantly, resveratrol directly activates SIRT7 deacetylase, thus antagonizing TGF-β signaling and inhibiting breast cancer lung metastasis. Our data provide SIRT7 as a novel anti-metastatic therapeutic target and resveratrol as a candidate molecule with potential anti-metastatic and anti-aging benefits.

The function of SIRT7 in tumor development is seemingly controversial in the literature. On one side, SIRT7 cooperates with Elk4 to maintain oncogenic transformation and tumor growth via H3K18ac deacetylation in local promoters^19^, and is correlated with poor prognosis in colorectal and prostate cancers^42, 43^. On the other side, Sirt7 are consistently downregulated in tumorigenic cells compared to non-tumorigenic ones in mice^44^, and SIRT7 is negatively correlated with progression of human head and neck squamous cell carcinoma^45^. Interestingly, Aljada et al.^46^ showed that SIRT7 is significantly upregulated in early stage of breast cancers but gradually declined with tumor progression. Indeed, oncogenic transformation, tumor growth, and metastasis are phenotypically distinct and mechanistically different processes. Ooms et al.^47^ have found that activation of AKT led to fast-growth breast cancer but with little metastasis in the PyMT model. Thus, it is possible that SIRT7 itself is dynamically regulated during tumor development, and at early stage, high SIRT7 promotes oncogenic transformation and tumor growth, but later on, it rather inhibits dissemination, migration, and invasion. Indeed, we found strong expression of SIRT7 in primary tumor site (Fig. 1a, d), and in primary breast cancer cell lines (Supplementary Fig. 2d, e). Moreover, we found *SIRT7* deficiency does not affect the proliferation capacity of in vitro cultured breast cancer cells, but promotes in vivo tumor growth in xenograft models (Supplementary Fig. 3). Together, these observations might attribute to the non-cell-autonomous neo-angiogenesis caused by activated TGF-β signaling^48^. Indeed, owing to intrinsic differences in tumor-initiating mechanism and tumor microenvironment, it has been shown that TGFβ blockade has little effect on PyMT-mediated breast cancer development but inhibits 4T1 tumor growth via increased tumor blood vessel perfusion^49^. Ding et al.^50^ found that SMAD4 constrains prostate cancer growth and metastatic progression and impaired SMAD4 expression in mouse model accelerated prostate cancer growth and metastasis. Given that we found SIRT7 destabilized SMAD4, it indirectly supports the notion that higher expression of SIRT7 promoted prostate cancer metastasis^43^. Breast cancers are more or less different from other cancers. Metastatic breast cancer cells escape the cytostatic response of TGF-β, and rather utilize SMAD4 to amplify TGF-β signaling and educate metastatic environment^11, 16^. Mechanistically, the seemingly discordance might attribute to pleotropic deacetylation targets of SIRT7 in different cell origins, such as H3K18^19^, p53^28^, PAF53^51^, NPM1^52^, GABP-β1^53^, and the overt phenotypes could attribute to combined effects of various targets. Together, our discovery provides new insight for TGFβ regulation during breast cancer metastasis, and we believe SIRT7 belongs to the key regulators that specifically targets breast cancer lung metastasis. It is very important because thus far effective therapeutic strategies targeting breast cancer metastases are still scarce.

SMAD4 is a core factor of TGF-β signaling, and has been identified as a common tumor suppressor in colon and pancreatic cancers, owing to aberrant protein degradation^54^. Here we found SIRT7 deacetylates SMAD4 at K428. The K428 acetylation enhances the formation and nuclear translocation of SMAD complex, while its deacetylation promotes dissociation of SMAD complex, nuclear exportation, and degradation of SMAD4 (Supplementary Fig. 12). It has been shown that acetyltransferase p300 interacts with SMAD4 and regulates TGF-β signaling^55^. In future study, it would be interesting to examine whether p300 mediates acetylation of K428. Nonetheless, we highlight the (de)acetylation regulation is critical for breast cancer progression and metastasis. Interestingly, it has been shown that loss of *Sirt1* causes hyper-acetylation of Smad4 and promotes breast cancer metastasis^56^, while another independent study demonstrated that Sirt1 deacetylase activity is dispensable for breast tumorigenesis and metastasis^57^. Recently, it has been shown that SIRT1 recruits SIRT7 to local promoter thus inhibiting gene transcription^43^. Thus, it is plausible to speculate that SIRT1 might regulate SMAD4 and breast cancer metastasis in a SIRT7 deacetylase-dependent manner. Moreover, we found resveratrol directly activates SIRT7 and thus to regulate SMAD4 deacetylation and stability, independent of SIRT1.

The crucial functions of TGF-β in cancer and other diseases have engaged numerous efforts on developing targeted therapeutics^58^. Current targeted strategies mainly focus on the ligands and/or receptors, e.g., TGF-β-neutralizing antibodies, receptor kinase inhibitors, and ligand trap^58^. Indeed, patients with late-stage cancers have got promising efficacies from TGF-β inhibitors in phase I/II clinical trials^59^. However, the major obstacles for TGF-β-targeted therapies are its spatial and temporal activation during cancer progression^60^. While TGF-β signaling antagonizes cell growth at early stage^61^, malignant breast cancer cells escape from this effect and become highly dependent on TGF-β factors to drive metastasis^62^. Conventional anti-cancer therapies such as radiation and chemotherapy may even increase circulating TGF-β1, promoting metastasis^63^. In addition, systemic anti-TGF-β therapies might bring severe side effects, such as inflammation, autoimmunity, or cardiovascular defects^64^. Therefore more specific drugs that selectively target downstream signaling without affecting other homeostatic functions of TGF-β would be an optimal choice. In our study, we found SIRT7 expression is significantly decreased in metastatic breast cancers to promote TGF-β signaling activation, which might be a suitable strategy to operate TGF-β signal during breast cancer progression. SIRT7-mediated deacetylation of SMAD4 could be utilized as a TGF-β blocker if targeted therapy is necessary in appropriate cancer patients. Further, targeting SIRT7-SMAD4 axis might have less adverse effects in comparison to TGF-β ligand or receptor, considering that SIRT7 is an important aging regulator and loss of expression accelerates aging in stem cells and mice^23, 28^. We also found resveratrol activates SIRT7 deacetylase activity toward SMAD4 to inhibit breast cancer lung metastasis. Our findings could inspire the development of drugs that selectively target SIRT7 in breast cancer metastasis, with potential anti-aging benefits.

## Methods

### Cell lines and immunofluorescence staining

Human breast cancer cell lines MDA-MB-231, BT549, T47D, and murine breast cancer cell line 4T1 were obtained from American Type Culture Collection (ATCC). HaCaT cells were purchased from the Chinese Academy of Sciences Committee Type Culture Collection Cell Bank (Shanghai, China). F2S human dermal fibroblast cells were a gift from D. Chan (School of Biomedical Sciences, The University of Hong Kong). MDA-MB-231 cells were cultured in α-MEM (Gibco) supplemented with 10% FBS (Gibco), non-essential amino acids, and sodium pyruvate (Gibco). BT549 cells were cultured in RPMI-1640 (Gibco) with 10% FBS. T47D, human fibroblast F2S, HaCaT, and murine breast cancer cell line 4T1 were maintained in DMEM (Gibco) with 4.5 g/L glucose and 10% FBS. To examine the EMT trait, TGF-β1 (5 ng/ml) were added to T47D cells before further analysis. All cells were authenticated by cell characteristics and were not contaminated by mycoplasma determined by PCR (Takara, Japan). For Immunofluorescence staining, cells on coverslips were fixed in 4% paraformaldehyde, permeabilized with 0.1% Triton X-100, and blocked with 1% BSA. The cells were then incubated with primary antibodies overnight at 4 °C, followed by secondary antibodies for 1 h at room temperature. Antibody information was listed in Supplementary Table 1.

### Cell transfection and RNA interference

Plasmid and siRNA transfection were performed with Lipofectamine3000 (Thermo) following the manufacturer’s instruction. Specific custom siRNAs were synthesized in GenePharma (Shanghai, China). For stable knockdown, shRNAs were cloned into pLKO.1 plasmid (Sigma). To obtain viral particles, 10 µg of lentiviral construct, 10 µg of pSPAX2, and 5 µg of pMD2G were co-transfected into HEK293T cells using Lipofectamine3000 (Thermo). About 48 h after transfection, the supernatants were collected and filtered through 0.22 µm membrane (Millipore). Virally infected cells were selected with appropriate concentration of puromycin (Sigma) to obtain stable cell lines. The sequences of siRNA or shRNA used in this study were listed in Supplementary Table 2.

### Wound healing and Transwell invasion assays

For wound healing assay, BT549, MDA-MB-231, or 4T1 cells were cultured in six-well plates coated with 0.1% gelatin. When 70% confluency is reached, the cells were starved overnight, wound was scratched in the center of the cell monolayer by a sterile plastic pipette tip, and debris was removed by PBS washing. The wound was photographed at indicated time. For Transwell invasion assay, 5 × 10^4^ cells suspended in medium without FBS were plated on the upper chamber membranes (8 µm pore size, 6.5 mm diameter, Corning) coated with Matrigel (BD Biosciences). The insert was incubated in 500 µl medium with 10% FBS or TGF-β1. To evaluate the invasive ability, non-invasive cells were removed by swiping the top of membrane with cotton swabs and invasive cells were stained with crystal violet and counted.

### RNA isolation and quantitative RT-PCR

Total RNA was isolated using Trizol reagent RNAiso Plus (Takara). Total mRNA was reversely transcribed into cDNA using the 5× Primescript RT Master Mix (Takara). Quantitative RT-PCR was performed using 2× SYBR Green Mix (Takara) in Bio-Rad detection system. The primers were listed in Supplementary Table 2.

### Western blotting and immunoprecipitation

Protein extracts for western blotting were prepared in Laemmli loading buffer (0.1 M Tris-HCl (pH 7.0), 4% SDS, 20% glycerol, 1 mM DTT, and protease inhibitors), then separated by SDS-polyacrylamide gels, transferred to PVDF membrane (Millipore) and probed with respective antibodies. Immunoblots were visualized by the Bio-Rad system. Antibody information was listed in Supplementary Table 1. For immunoprecipitation, cells with indicated treatments were lysed in 200 mM KCl, 20 mM Tris-HCl (pH 7.9), 5 mM MgCl_2_, 10% glycerol, 0.2 mM EDTA, and 0.1% NP-40, supplemented with protease inhibitors (Roche Complete). Clear cell lysates were then incubated with the respective antibodies or control IgGs at 4 °C overnight. Beads-bound immunoprecipitates were washed, eluted in Laemmli loading buffer, and analyzed by western blotting. Uncropped scan of immunoblots was shown in Supplementary Fig. 13.

### Protein pull down assay

For GST-pull down, 1 µg of GST or GST-SIRT7 protein was immobilized on Glutathione-Sepharose 4B (GE), incubated with 6× His-tagged SMAD4 purified from bacterial culture for 2 h at 4 °C in 120 mM KCl, 20 mM Tris-HCl (pH 7.9), 5 mM MgCl_2_, 0.2 mM EDTA, 10% glycerol, 0.2% NP-40, and protease inhibitors (Roche Complete). Beads were washed and then analyzed by western blotting. His-tagged-pull down assay was performed by Ni-NTA agarose (Qiagen) through the similar method mentioned above.

### In vitro deacetylation assay

In vitro deacetylation assay was performed as described^51^. Briefly, HEK293T cells expressing HA-SMAD4 were pre-treated with HDAC inhibitors (10 mM NAM, 50 nM TSA, 5 mM Sodium butyrate) for 6 h, then lysed in 300 mM KCl, 20 mM Tris-HCl (pH 7.9), 5 mM MgCl_2_, 0.2 mM EDTA, 10% glycerol, 0.5 mM DTT supplemented with 0.1% NP-40, protease inhibitors (Roche) and HDAC inhibitors (Sigma). HA-SMAD4 was purified with anti HA resin affinity (Sigma). For in vitro deacetylation, beads-bound HA-SMAD4 was incubated with 1 µg of GST-SIRT7 in 100 mM KCl, 20 mM Tris-HCl (pH 7.9), 5 mM MgCl_2_, 0.2 mM EDTA, 10% glycerol, 0.5 mM DTT in the presence or absence of 5 mM NAD^+^ at 30 °C for 1 h. Acetylation level of SMAD4 was monitored by western blotting with anti-pan-acetylation lysine antibodies.

### Ubiquitin assay

HEK293T cells were transfected with Myc-ubiquitin construct together with indicated plasmids. After 48 h, cells were harvested in immunoprecipitation buffer mentioned above by sonication. The ubiquitination of indicated proteins was immunoprecipitated and analyzed by western blotting with anti-ubiquitin antibody.

### Breast cancer survival analysis

Kaplan–Meier survival analyses for clinical outcomes (RFS or DMFS) of breast cancers were performed using web tool Kaplan–Meier Plotter (http://kmplot.com/analysis/)^27^, cBioPortal (http://www.cbioportal.org)^65, 66^, and PROGgene V2^24^. The percentiles of the patients between the upper and lower quartile were auto-selected based on the best performing thresholds as cutoffs.

### CRISPR/Cas9 genome editing

pX459 vector (Addgene#48139) was digested with *Bbs*I and ligated with indicated annealed oligonucleotides: hSIRT7 g-F: 5′-CACCGTGTGTAGACGACCAAGTATT-3′, hSIRT7 g-R: 5′-AAACAATACTTGGTCGTCTACACAC-3′, hSIRT1 g-F: 5′-CACCGATAGCAAGCGGTTCATCAGC-3′, hSIRT1 g-R: 5′-AAACGCTGATGAACCGCTTGCTATC-3′, HEK293T cells and BT549 breast cancer cells were transfected with pX459-SIRT7 and pX459-SIRT1 construct, respectively. Potential mutant cells clones were picked after 5 days selection with 1 µg/ml puromycin and confirmed by western blotting and genome sequencing (BGI).

### Gene set enrichment analysis

GSEA was performed with GSEA v2.0 on various functional and/or characteristic gene signatures^33, 67^. Gene sets were obtained from published gene signatures as mentioned in manuscript. Statistical significance was assessed followed as the published method^68^.

### Transgenic mouse models

Murine mammary cancer model FVB/N-Tg (MMTV-PyMT)/Nju was purchased from Model Animal Research Center of Nanjing University. Tet-on *Sirt7* transgenic mice (*Sirt7* ^tg^) were obtained through transgenic animal services from Cyagen, Guangzhou, China. PyMT mice were crossed to *Sirt7* ^tg^ to get PyMT and PyMT; *Sirt7* ^tg^ females. To study the tumor initiation and lung metastasis, mice at the age of 5 weeks were assigned randomly to two experimental groups. One group was fed with doxycycline (2 mg/ml) in drinking water and the other group with water only. We did not perform experiments in a blind manner. After 16 weeks, all mice were killed and lung metastasis was analyzed. All mice were housed and handled in accordance with protocols approved by the Committee on the Use of Live Animals in Teaching and Research of Shenzhen University. To minimize suffering, all surgeries were performed under anesthesia.

### Xenograft model of breast cancer lung metastasis

Pathogen-free female BALB/c and athymic nude mice were purchased from the Slaccas (Shanghai). All mice were housed and handled in accordance with protocols approved by the Committee on the Use of Live Animals in Teaching and Research of Shenzhen University. We did preliminary experiments to determine the need for mice sample size. All mice were assigned randomly to experimental groups and we did not perform in a blind manner. MDA-MB-231 cells (1 × 10^6^) were injected into the lateral tail vein of athymic nude mice. 4T1 cells (5 × 10^5^) were injected into the fourth mammary fat pad of virgin BALB/c mice. Mice were killed on day 35 after tail vein injection or on day 28 after mammary fat pad injection. The number of metastatic nodules on the surface of lung was counted under dissecting microscope after H&E staining. For resveratrol treatment, BALB/c mice were inoculated with 5 × 10^5^ 4T1 cells in the fourth mammary fat pad. About 7 days after inoculation, tumor-bearing mice were randomized for treatment with saline or resveratrol (100 mg/kg, i.p.) every other day and killed on day 28 for lung metastasis examination. Tumor diameters and mouse weight were monitored four times weekly. Tumor volume (mm^3^) was calculated by the formula: Volume = 0.5 × length × width^2^.

### Human breast cancer tissue array analysis

Human breast cancer tissue arrays (BR963c and BR10010d) were bought from Cybrdi (Shanxi Chao Ying Biotechnology, China). Pathological diagnosis was provided in the manufacturer’s instructions. Paraffin-embedded sections from primary breast tumors and paired lung metastases were obtained from Shenzhen Second People’s Hospital, Shenzhen University. All human breast cancer sample acquisitions were approved by the Committee on Ethics of Shenzhen Second People’s Hospital, Shenzhen University. Written informed consent was obtained from all the participants. Immunohistochemical staining was performed as follows. Negative control slides without primary antibodies were included. The core staining was scored as negative (0) when <10% tumor cells showed expression. Positive scores (1+ to 3+) were based on percent of tumor cells combining positive staining intensity within the tumor sample. For IHC score distribution analysis, unpaired *t*-test with Welch’s correction was applied for comparison of different type of tissues. In *χ* ^2^ analysis, scores are defined as follows: low = 0/1+, high = 2+/3+. IHC staining was performed in a blind manner and pathologists who performed the IHC staining and scoring did not know the original hypothesis.

### Statistical analysis

All experiments were carried out with at least three replicates. The data were shown as mean ± S.D. or mean ± S.E.M. as indicated in the figure legends. For comparison of central tendencies, normally distributed data sets were analyzed by unpaired two-sided Student’s *t*-tests under assumption of equal variance; non-normally distributed data sets were analyzed by non-parametric Mann–Whitney *U*-tests. *χ* ^2^-test was applied to analyze the relationship between SIRT7 levels and pathological status. Differences were considered as statistically significant when *P* < 0.05.

### Data availability

The Kaplan–Meier survival analyses and SIRT7-related signaling pathway predication data sets used in this study are available as described in the manuscript. Data supporting the finding of this study are available from the Gene Expression Omnibus (accession no. GSE99596) or from the corresponding author upon reasonable request.

## Electronic supplementary material

Supplementary Information

Supplementary Data 1

Supplementary Data 2

Supplementary Data 3

Supplementary Data 4
