# Supplementary material for: SIRT7 antagonizes TGF-β signaling and inhibits breast cancer metastasis
Source: Nat Commun. 2017 Aug 22;8:318. doi: 10.1038/s41467-017-00396-9 (PMC5566498; doi:10.1038/s41467-017-00396-9)
Supplement: Supplementary file 1 — Supplementary Information [file 41467_2017_396_MOESM1_ESM.pdf]

File Name: Supplementary Information
Description: Supplementary Figures, Supplementary Tables and Supplementary References

File Name: Supplementary Data 1
Description: Survival analysis of breast cancers. Kaplan-Meier plot analysis of recurrence free survival (RFS), distant metastasis free survival (DMFS) and lung metastasis free survival (LMFS) based on indicated genes, dataset and analysis tool as previous description.

File Name: Supplementary Data 2
Description: Clinicopathologic parameters of lymph node metastases and paired primary breast cancers, related to Figure 1

File Name: Supplementary Data 3
Description: Differentially expressed genes in SIRT7 KD BT549 cells

File Name: Supplementary Data 4
Description: Clinicopathologic parameters of breast cancers, related to Figure 7

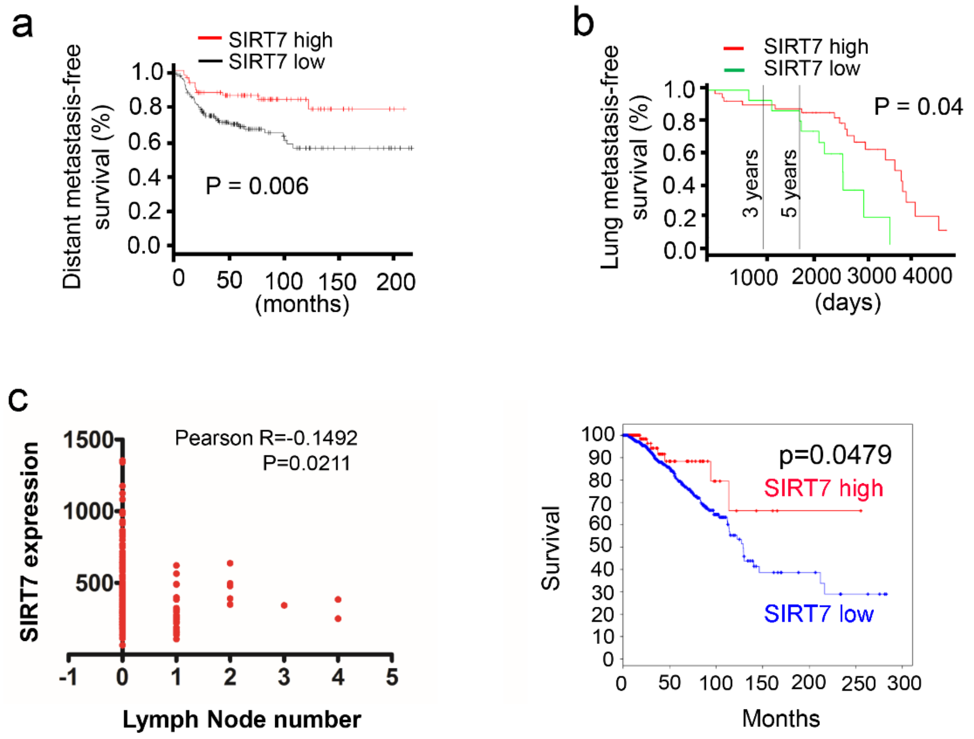

Supplementary Figure 1. SIRT7 predicts favorable prognosis of breast cancers

(**a**) Distant metastasis-free survival (DMFS) of breast cancers, stratified by SIRT7 level, in Kaplan-Meier database[1]. (**b**) Lung metastasis-free survival of breast cancers based on SIRT7 level in GSE5237 dataset[2]. (**c**) SIRT7 level was negatively correlated with progressive lymph node metastasis and poor prognosis a cohort of breast invasive carcinoma[3].

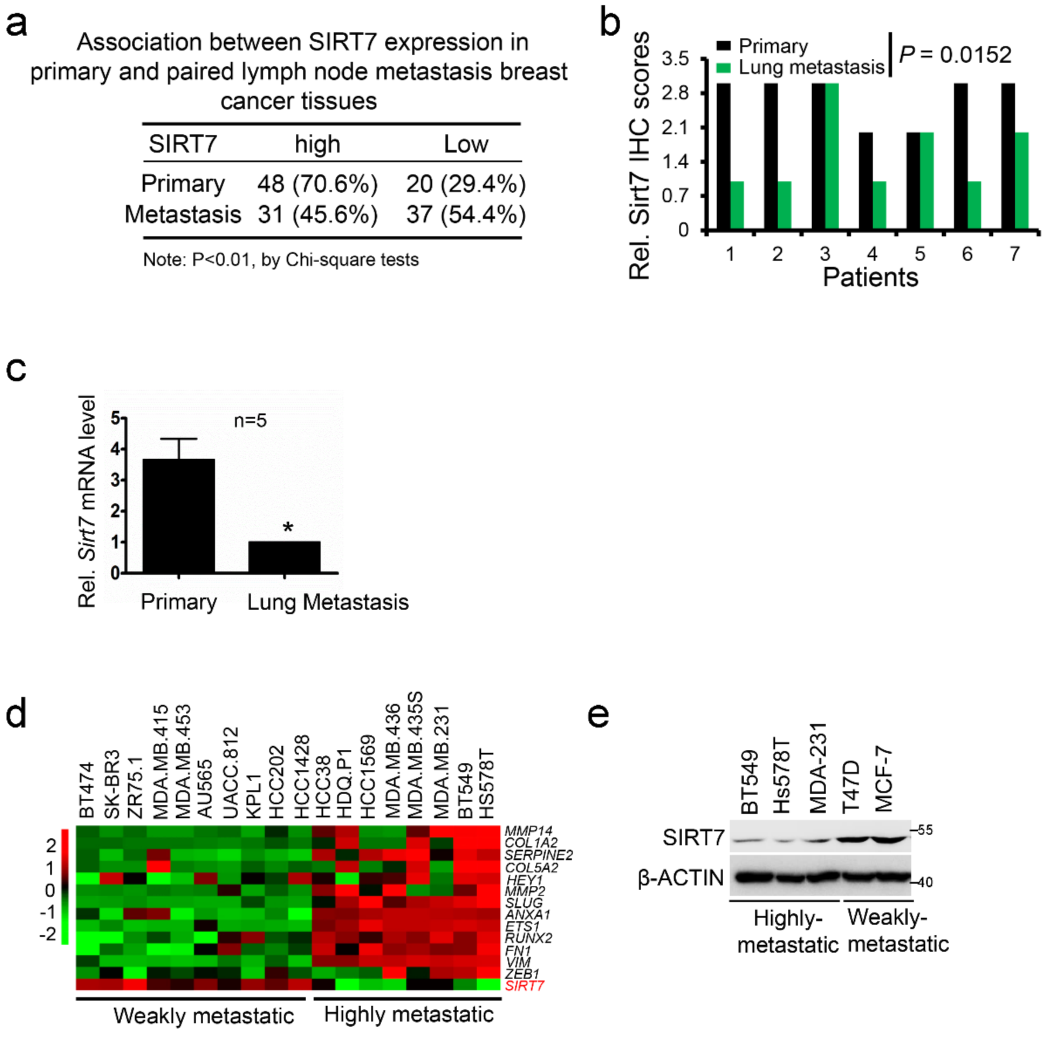

Supplementary Figure 2. SIRT7 is associated with breast cancer metastasis

(**a**) Association between SIRT7 expression in primary and lymph node metastasis breast cancer tissues. (**b**) SIRT7 IHC scores in primary and paired lung metastasis breast cancers. *P* value was determined by paired Student's *t*-test. (**c**) Quantitative RT-PCR analysis of SIRT7 mRNA level in breast cancer cells isolated in Figure 1f. (**d**) Heatmap showing relative expression levels of SIRT7 and other metastasis-related genes in various breast cancer cell lines with different metastatic abilities. Data were obtained from published dataset[4,5]. (**e**) Immunoblot analysis of SIRT7 in breast cancer cell lines with different metastatic ability. $^{*}P < 0.05$, $^{**}P < 0.01$. *P* value was analysed by Chi-square test (a), Student's *t*-test (b, c). Data are shown as mean ± S.E.M.

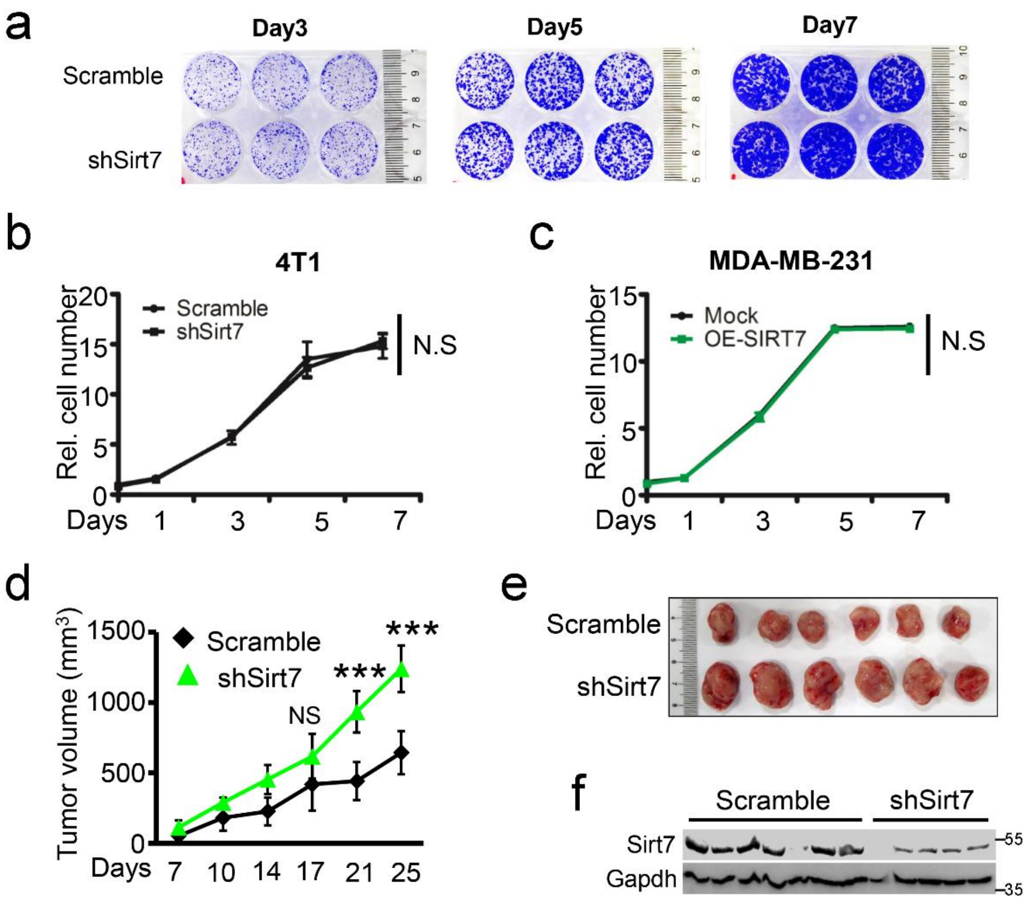

Supplementary Figure 3. Effect of SIRT7 on cell and xenograft tumor growth

(**a**) Colony formation assay of Scramble and shSirt7-expressing 4T1 cells. (**b**-**c**) Growth curve of *Sirt7* KD 4T1 cells (**b**) and ectopic *SIRT7*-expressing MDA-MB-231 cells (**c**). (**d**) Tumor growth of Scramble and shSirt7-expressing 4T1 cells. (**e**) Photographs of tumors in (**d**). (**f**) Immunoblots showing *Sirt7* expression in tumors (**e**). ***$P < 0.001$. P value is calculated by Student's t-test. Data are mean ± S.E.M.

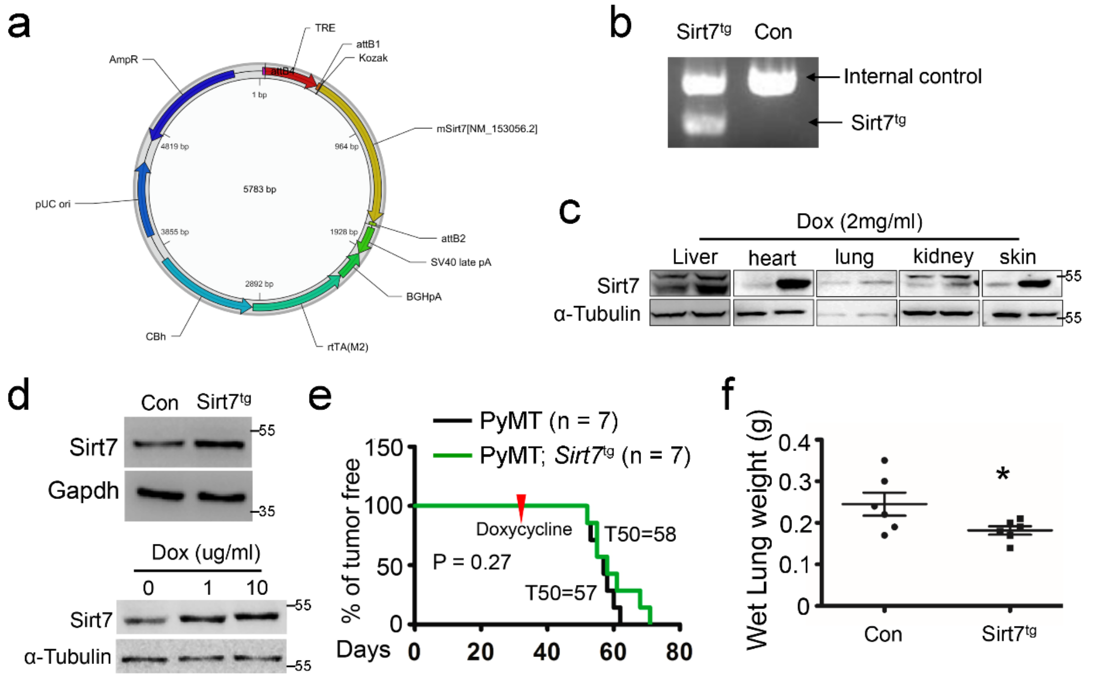

Supplementary Figure 4. ***Sirt7*** **transgenic mice and tumor development**

(**a**) Schematic map of construct used for transgenic microinjection. (**b**) Genotyping analysis of *Sirt7*^{tg} mice. (**c**) Immunoblotting analysis of *Sirt7* expression in various tissues of *Sirt7*^{tg} mice after feeding with doxycycline (2 mg/ml) for 3 days. (**d**) Immunoblots of Sirt7 in primary tumors (upper) and cells (lower) isolated from PyMT; *Sirt7*^{tg} or control mice. (**e**) Kaplan-Meier curve showing latency of tumor induction in PyMT and PyMT; *Sirt7*^{tg} mice fed with Dox. T50, days of 50% of mice burden with palpable tumor masses. *P* value was calculated by the log rank test. (**f**) Scatter plot showing the wet lung weight of mice in (**e**). *$P < 0.05$, calculated by Student's *t*-test (**f**).

a

b

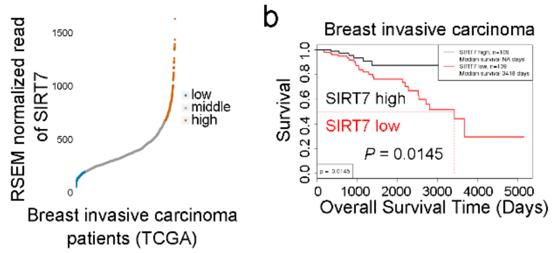

c

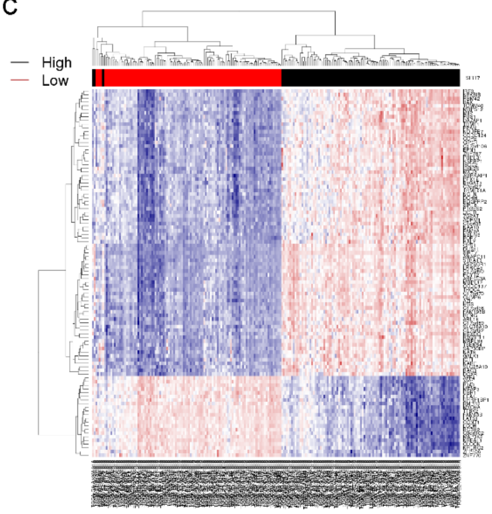

d SIRT7 negatively associated genes

Pathway	p.geomean	stat.mean	p.val	q.val	set.size
**hsa04510 Focal adhesion**	**0.00011**	**-3.83625**	**1E-04**	**0.01**	**55**
**hsa04512 ECM-receptor interaction**	**0.00035**	**-3.68957**	**4E-04**	**0.01**	**28**
hsa04972 Pancreatic secretion	0.00177	-3.39427	0.0018	0.035	15
hsa04630 Jak-STAT signaling pathway	0.00221	-3.00039	0.0022	0.035	27
hsa04020 Calcium signaling pathway	0.00254	-2.8989	0.0025	0.035	36
hsa04360 Axon guidance	0.00306	-2.84102	0.0031	0.035	32
**hsa04350 TGF-beta signaling pathway**	**0.00356**	**-2.90561**	**0.004**	**0.04**	**21**
hsa04810 Regulation of actin cytoskeleton	0.00406	-2.70267	0.0041	0.035	52
hsa04270 Vascular smooth muscle contraction	0.00698	-2.53492	0.007	0.054	31
hsa02010 ABC transporters	0.01495	-2.34241	0.015	0.103	13
hsa04540 Gap junction	0.03588	-1.84057	0.0359	0.201	26
hsa04974 Protein digestion and absorption	0.03743	-1.83086	0.0374	0.201	21
hsa00510 N-Glycan biosynthesis	0.03864	-1.87511	0.0386	0.201	10
hsa00562 Inositol phosphate metabolism	0.04081	-1.82046	0.0408	0.201	14
hsa04310 Wnt signaling pathway	0.04824	-1.69809	0.0482	0.215	24
hsa04970 Salivary secretion	0.04995	-1.702	0.05	0.215	15

Supplementary Figure 5. SIRT7 expression is associated with TGF-β signaling

(**a-b**) Overall distribution of *SIRT7* level (**a**) and survival analysis (**b**) in the TCGA RNAseq dataset. (**c-d**) Pathway enrichment by KEGG according to SIRT7 expression. Noted a significant correlation between low level of SIRT7 and TGF-β signaling.

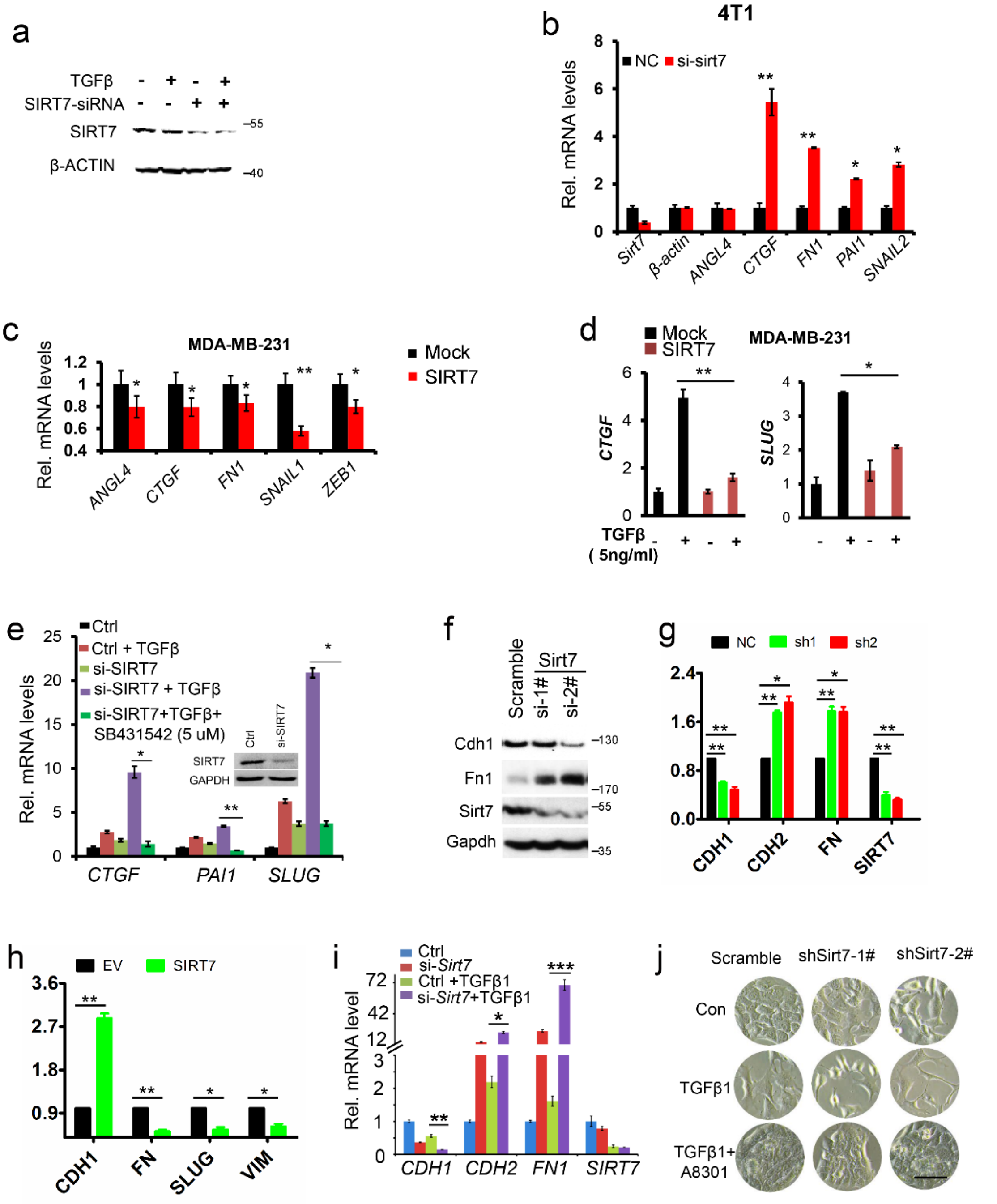

Supplementary Figure 6. Loss of SIRT7 promotes EMT in breast cancer cells

(**a**) Western blotting analysis of SIRT7 in BT549 cells treated with Scramble or siSIRT7. (**b**) Quantitative RT-PCR analysis of TGF-β downstream and EMT-related genes in 4T1 cells treated with Scramble or si-*SIRT7*. (**c**) Quantitative RT-PCR analysis of TGF-β downstream and EMT-related genes in MDA-MB-231 cells expressing ectopic *SIRT7*. (**d**) Quantitative RT-PCR analysis of TGF-β downstream gene *CTGF* and *SLUG* in

MDA-MB-231 cells expressing ectopic *SIRT7* in the presence or absence of TGF-β1 (5 ng/ml, 2 h). (**e**) Quantitative RT-PCR analysis of TGF-β downstream gene *SLUG*, *PAI1*, *CTGF* levels in cells treated with TGFβ1 (5 ng/ml) or TGFβR1 inhibitor SB431542 (5 μM). (**f**) Western blotting analysis of Sirt7 and EMT markers in 4T1 cells treated with Scramble or *Sirt7* siRNAs. (**g-h**) Quantification of immunoblots in Figure 3j and 3k, respectively. (**i**) Quantitative RT-PCR analysis of EMT markers in *SIRT7* KD T47D cells under the stimulation of 5 ng/ml TGF-β1 for 48 h. (**j**) Phase contrast images showing morphological changes in T47D cells under the indicated treatments. Scale bar, 50 μm. *$P < 0.05$, **$P < 0.01$. P value is analysed by Student's *t*-test. Data are shown as mean ± S.E.M. and representative of two or three independent experiments.

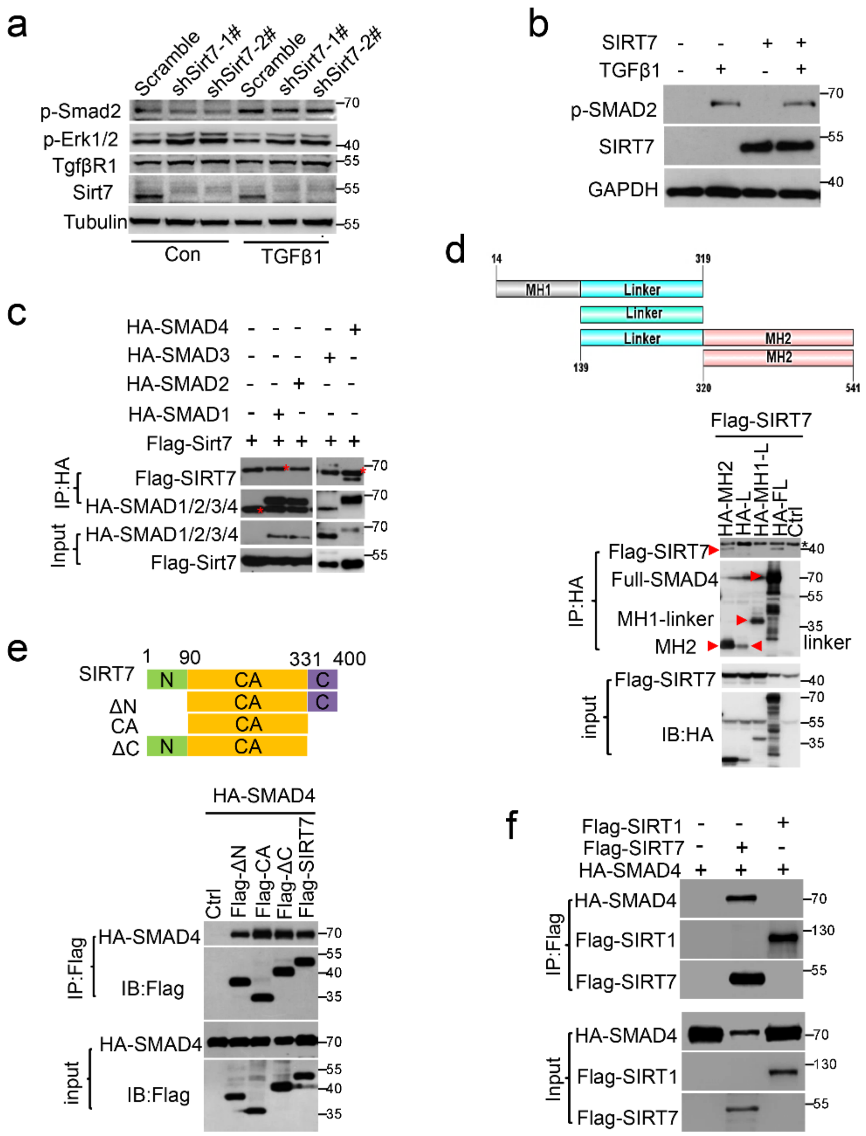

Supplementary Figure 7. SIRT7 interacts with SMAD4

(**a-b**) Western blotting analysis of TGF-β pathway components in *Sirt7* KD 4T1 cells (**a**) and BT549 cells expressing ectopic SIRT7 (**b**), in the presence or absence of TGF-β1 (5 ng/ml). (**c**) Immunoblots showing SIRT7 in anti HA-SMAD4 immunoprecipitates, but not in anti HA-SMAD1/2/3 immunoprecipitates. Asterisks indicate IgG heavy chain. (**d**) Upper, schematic illustration of various SMAD4 mutants[6]. Lower, SIRT7 was only detected in anti HA-FL (full length SMAD4) and anti HA-MH2 (MH2 domain of SMAD4) immunoprecipitates. Triangles indicate different HA-SMAD4 mutants and asterisk refers to IgG heavy chain. (**e**) Upper, schematic illustration of SIRT7 mutants used for SMAD4 co-immunoprecipitation, i.e. N-terminal deleted (Flag-ΔN), catalytic domain (Flag-CA), C-terminal deleted (Flag-ΔC) and full length SIRT7. Lower,

Western blotting analysis of HA-SMAD4 in the anti SIRT7 mutant immunoprecipitates. (**f**) Immunoblots showing interaction between SMAD4 and SIRT7, but not SIRT1.

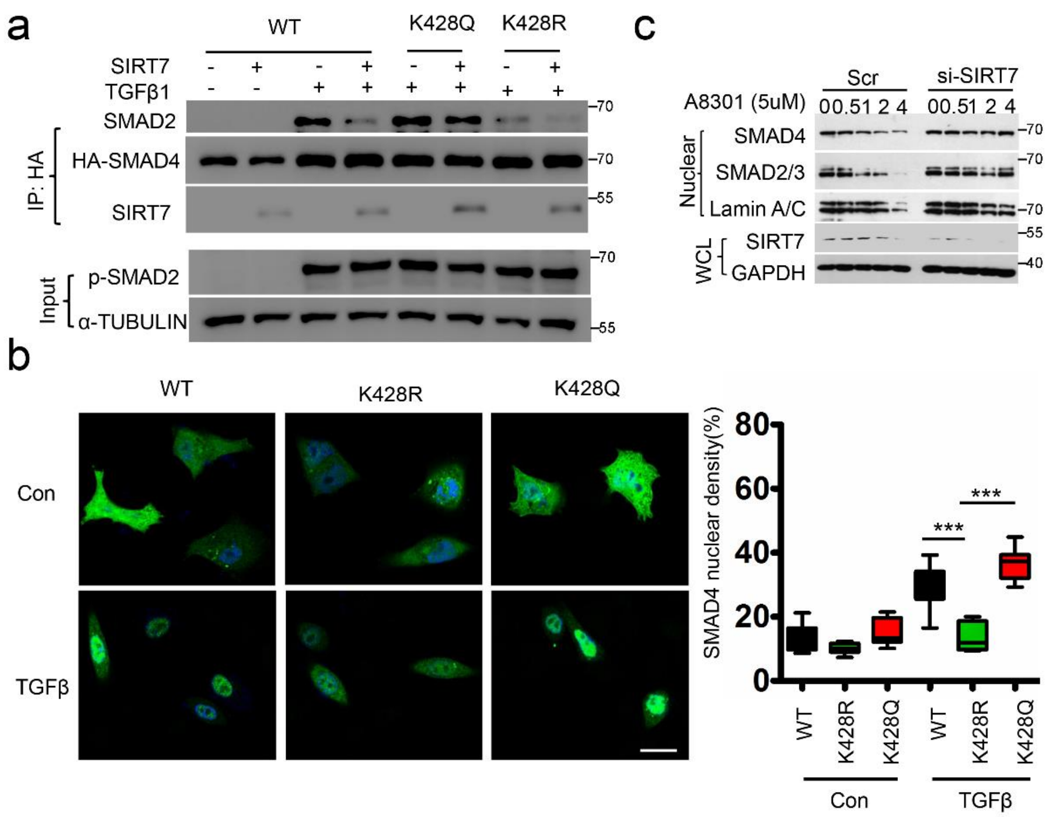

Supplementary Figure 8. SMAD complex formation and nuclear-cytoplasmic shuffling

(**a**) MDA-MB-231 cells expressing wild-type SMAD4, K428R or K428Q mutants were treated with TGF-β1 (5 ng/ml) for 30 minutes before anti SMAD4 immunoprecipitation. Noted less SMAD2 in anti SMAD4 K428R immunoprecipitates, but more SMAD2 in anti SMAD4 K428Q immunoprecipitates compared with wild-type SMAD4. (**b**) Left, Immunofluorescence staining of SMAD4 mutants in cells treated with TGF-β1 (5 ng/ml) for 2 h. Scale bar, 50 μm. Right, box plots showing the percentage of nuclear intensity of SMAD4. 100 cells were randomly selected and quantified by Image J®. (**c**) *SIRT7* KD HaCaT cells were treated with TGF-β1 (5 ng/ml) for 30 minutes, then washed to remove TGF-β1, treated with A8301 (5 μM) for indicated time, and nuclear fractions were collected. Representative Immunoblots showing dynamic levels of SMADs in nucleus. ***$P < 0.001$, calculated by Student's t-test. Data are mean ± S.E.M.

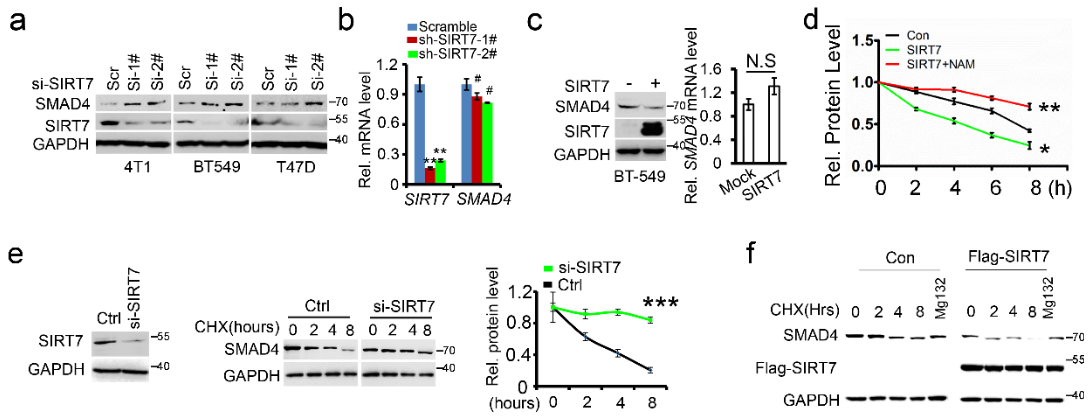

Supplementary Figure 9. SIRT7 destabilizes SMAD4 protein

(**a**) Immunoblots showing SMAD4 protein levels in three breast cancer cell lines treated with Scramble or *SIRT7* siRNAs. (**b**) Quantitative RT-PCR analysis of *SMAD4* mRNA levels. (**c**) Western blotting and real-time PCR analyses of SMAD4 expression levels in BT549 cells expressing ectopic SIRT7 or empty vector. (**d**) Quantification of Figure 6b. (**e**) Immunoblots showing endogenous SMAD4 protein levels in BT549 cells treated with or without *SIRT7* siRNA in the presence of CHX (50 μg/ml). (**f**) Immunoblots showing SMAD4 protein levels in control or SIRT7-overexpressing BT549 cells treated with CHX (50 μg/ml) and/or MG132 (10 μM). $*P < 0.05$, $**P < 0.01$, $***P < 0.001$. *P* values are calculated by one-way analysis of variance (ANOVA). N.S, no significance. Data are shown as mean ± S.E.M.

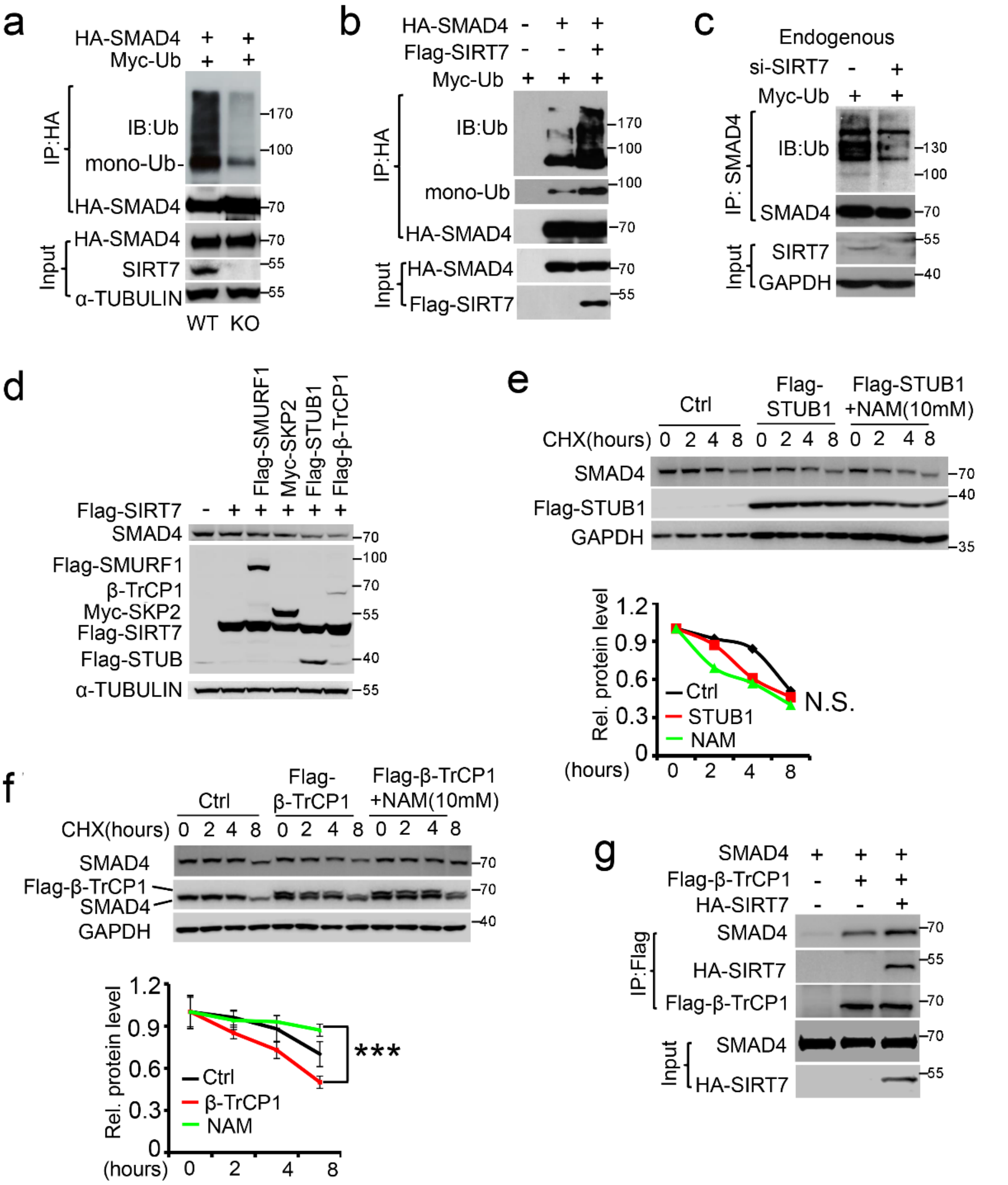

Supplementary Figure 10. SIRT7 promotes SMAD4 protein instability

(**a-b**) Western blotting analysis of HA-SMAD4 polyubiquitination in HEK293T cells lacking *SIRT7* (**a**) or expressing ectopic SIRT7 (**b**). Noted mono-ubiquitinated SMAD4. (**c**) Western blotting analysis of SMAD4 (endogenous) ubiquitination level in BT549 cells treated with or without SIRT7 siRNA. (**d**) Immunoblots showing SMAD4 levels in HEK293T cells transfected with SIRT7 and indicated E3 ligases. (**e-f**) HEK293T cells expressing Flag-β-TrCP1 (**e**) or Flag-STUB1 were treated with or without NAM (10 mM). SMAD4 protein levels were determined by Western blotting. Quantification

was performed by Image J®. (**g**) Immunoblots showing increased binding capacity of SMAD4 to β-TrCP1 in the presence of ectopic SIRT7. ***P < 0.001. P value is calculated by one-way analysis of variance (ANOVA). N.S. means no significance. Data are shown as mean ± S.E.M.

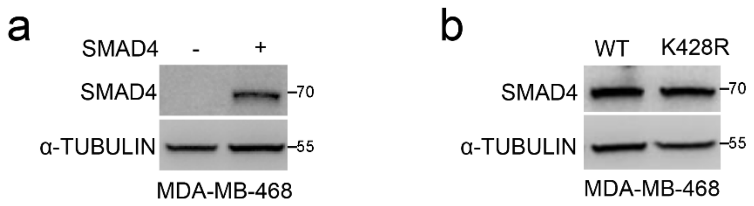

Supplementary Figure 11. Expression SMAD4 or K428R mutant

(**a**) Western blotting analysis of SMAD4 in *SMAD4* null MDA-MB-468 breast cancer cells as indicated treatment. (**b**) Western blotting analysis of WT SMAD4 and K428R in MDA-MB-468 cells.

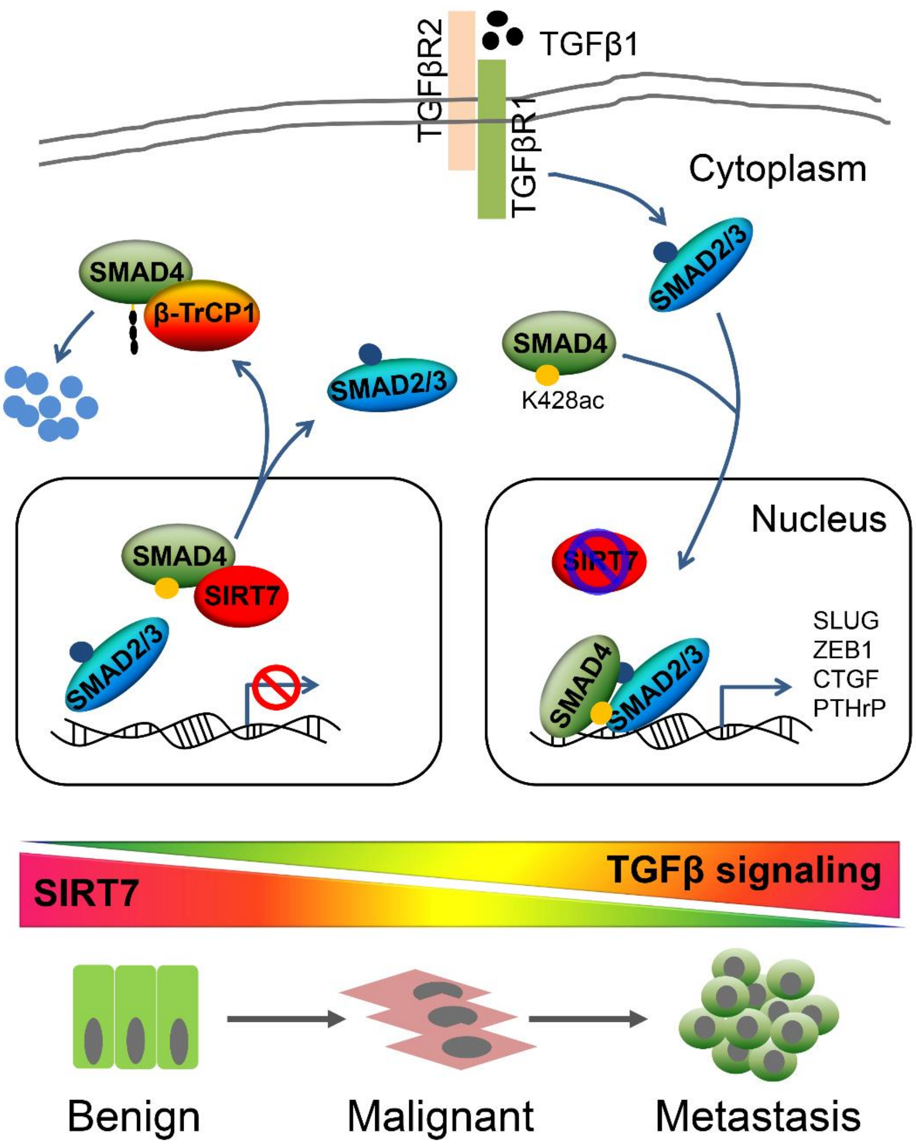

Supplementary Figure 12. Schematic model of SIRT7 in regulating breast cancer metastasis

Extracellular TGF-β1 activates TGFβR, which subsequently phosphorylates R-SMADs, i.e. SMAD2/3. Phosphorylated SMAD2/3 form heterotrimeric complex with co-SMAD, i.e. SMAD4. The K428 acetylation facilitates the formation and nuclear translocation of SMAD complex. SMADs corporately activate transcription of *SLUG*, *ZEB1*, *CTGF* and *PTHrP*, thus promoting EMT and cell migration and invasion. SIRT7 deacetylates SMAD4 at K428, promoting its dissociation from SMAD complex, nuclear exportation and subsequent degradation mediated by β-TrCP1. During breast cancer progression, gradually down-regulated SIRT7 leads to hyper-activation of TGF-β signaling, EMT and metastasis at later stage.

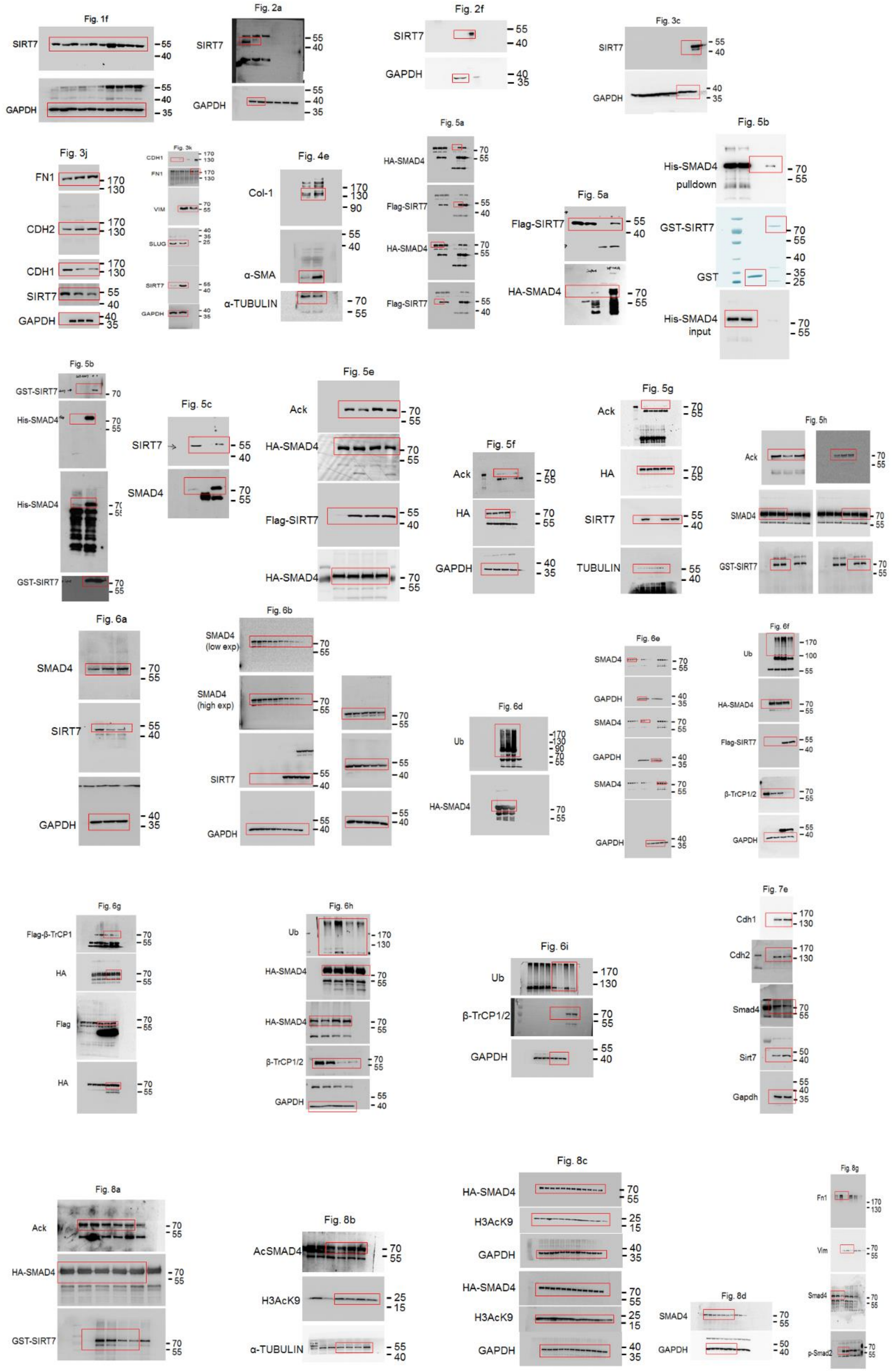

Supplementary Figure13. Uncropped scans of immunoblots

Antibody	Source	Dilutions
Ms α SMAD4	Santa Cruz (sc-7966)	WB/IF/IHC (1:1000/ 1:50/1:25)
Rb α acetyl lysine	Abcam (ab21623)	WB (1:250)
Ms α β-ACTIN	Beyotime (AA128)	WB (1:5000)
Ms α E-CDH1	BD Biosciences (610181)	WB/IF (1:5000/1:100)
Rb α SIRT7	Santa Cruz (sc-135055)	WB (1:2500)
Ms α N-Cadherin	BD Biosciences (610920)	WB (1:5000)
Rb α Fibronectin	Abcam (ab2413)	WB/IF (1:5000/1:100)
Rb α Vimentin	CST (3932S)	WB (1:1000)
Ms α Ubiquitin	CST (3936P)	WB (1:2500)
Rb α Erk1/2(pT202/T204)	CST (4370S)	WB (1:1000)
Rb α β-TrCP1	CST (4394S)	WB (1:1000)
Rb α Smad2/3	CST (5678S)	WB (1:1000)
pSmad2(Ser465/467)/ Smad3(Ser423/425)	CST (8828S)	WB (1:1000)
Rb α Phospho-Akt (ser473)	CST (9271S)	WB (1:1000)
Rb α Akt	CST (9272S)	WB (1:1000)
Rb α Slug	CST (9585S)	WB (1:500)
Rb α TGFβRI	Santa Cruz (sc-9048)	WB (1:500)
Ms α HA	Sigma-Aldrich (H3663)	WB (1:5000)
Ms α Tubulin	Beyotime (AT819)	WB (1:5000)
Ms α GAPDH	Beyotime (AG019)	WB (1:5000)
goat anti-mouse IgG	Jackson (11-035-003)	WB (1:10000)
goat anti-rabbit IgG	Jackson (15-035-003)	WB (1:10000)
Rb α SIRT1	Abcam (ab12193)	WB (1:1000)
Rb α SIRT7	EMD Millipore (ABE103)	IHC (1:50)
Ms α Flag	Sigma-Aldrich (F3165)	WB/IF/IP (1:5000/1:100)

Supplementary Table 1. Antibodies used in this study.

Targets	Sequence (5’-3’)	purpose
hSIRT7	TAGCCATTTGTCCTTGAGGA	shRNA
hSIRT7	CACCTTTCTGTGAGAACGGAA	shRNA
hSIRT7	CUCACCGUAUUUCUACUACUA	siRNA
mSirt7	TGCATCCCTAACAGAGAGTAT	shRNA
mSirt7	CCTCCCTCTTTCTACTCCTTA	shRNA
mSirt7	UGCAUCCCUAACAGAGAGUAU	siRNA
mSirt7	CCUCCCUCUUUCUACUCCUUA	siRNA
hSMAD4	GUACUUCAUACCAUGCCGA	siRNA
hSMAD4	CCAGCTACTTACCATCATA	shRNA
hSMAD4	GTACTTCATACCATGCCGA	shRNA
hSMAD7-F	CCCCATCACCTTAGCCGACTCTGC	qRT-PCR
hSMAD7-R	CCCAGGGGCCAGATAATTCGTTCC	qRT-PCR
hID2-F	TCAGCCTGCATCACCAGAGA	qRT-PCR
hID2-R	CTGCAAGGACAGGATGCTGAT	qRT-PCR
hGAPDH-F	TTGGTATCGTGGAAGGACTCA	qRT-PCR
hGAPDH-R	TGTCATCATATTTGGCAGGTT	qRT-PCR
h-SLUG-F	TGGTTGCTTCAAGGACACAT	qRT-PCR
h-SLUG-R	GCAAATGCTCTGTTGCAGTG	qRT-PCR
mβ-Actin-F	CCAGTTGGTAACAATGCCATGT	qRT-PCR
mβ-Actin-R	GGCTGTATTCCCCTCCATCG	qRT-PCR
hSIRT1-F	TACCGAGATAACCTTCTGTTCG	qRT-PCR
hSIRT1-R	GTTCGAGGATCTGTGCCAAT	qRT-PCR
hSIRT2-F	AGAAGCAGACATGGACTTCCT	qRT-PCR
hSIRT2-R	CTCCCACCAAACAGATGAC	qRT-PCR
hSIRT3-F	CATTCCAGACTTCAGATCGC	qRT-PCR
hSIRT3-R	AGCAGCCGGAGAAAGTAGT	qRT-PCR
hSIRT4-F	TGGGATCATCCTTGCAGGTAT	qRT-PCR
hSIRT4-R	TGGTCAGCATGGGTCTATCA	qRT-PCR
hSIRT5-F	GCCAAGTTCAAGTATGGCAGA	qRT-PCR
hSIRT5-R	CGCCGGTAGTGGTAGAA	qRT-PCR
hSIRT6-F	CAAGTGTAAGACGCAGTACGT	qRT-PCR
hSIRT6-R	ATGTACCCAGCGTGATGGAC	qRT-PCR
hSIRT7-F	ATGAGCAGAAGCTGGTGC	qRT-PCR
hSIRT7-R	CTGTCTGGTGTCTGTGGA	qRT-PCR
hβ-Actin-F	AGAGCTAGCTGCCTGAC	qRT-PCR
hβ-Actin-R	GGATGCCACAGGACTCCA	qRT-PCR
hPAI1-F	ATTCAAGCAGCTATGGGATTCAA	qRT-PCR
hPAI1-R	CTGGACGAAGATCGCGTCTG	qRT-PCR
hANGPTL4-F	TCCTGGGACGAGATGAATGTC	qRT-PCR
hANGPTL4-R	CTGAGCCTTGAGTTGTGTCTG	qRT-PCR
hCTGF-F	ACTGTCCCGGAGACAATGAC	qRT-PCR

hCTGF-R	TGCTCCTAAAGCCACACCTT	qRT-PCR
hCDH1-F	TGCCCAGAAAATGAAAAAGG	qRT-PCR
hCDH1-R	GTGTATGTGGCAATGCGTTC	qRT-PCR
hCDH2-F	ACAGTGGCCACCTACAAAGG	qRT-PCR
hCDH2-R	CCGAGATGGGGTTGATAATG	qRT-PCR
hFN-F	CAGTGGGAGACCTCGAGAAG	qRT-PCR
hFN-R	TCCCTCGGAACATCAGAAAC	qRT-PCR
hSMAD4-F	AAGCCATTGAGAGAGCAAGGT	qRT-PCR
hSMAD-R	GGTCACTAAGGCACCTGACC	qRT-PCR
mSmad7-F	CAGCACTGCCAAGCATGGT	qRT-PCR
mSmad7-R	ACCGAAACGCTGATCCAAAG	qRT-PCR
mβ-actin-F	ACTCCAAGGCCACTTATCACC	qRT-PCR
mβ-actin-R	ATTGTTACCAACTGGGACGACA	qRT-PCR
mSirt1-F	GTTGCCACCAACACCTCTTC	qRT-PCR
mSirt1-R	GCAGTACTGGAACCAACAGC	qRT-PCR
mSirt7-F	CCTGCATCCCTAACAGAGAG	qRT-PCR
mSirt7-R	AGCTGGACCCTAAACACAGG	qRT-PCR
hα-SMA-F	TCAATGTCCCAGCCATGTAT	qRT-PCR
hα-SMA-R	CAGCACGATGCCAGTTGT	qRT-PCR
mCol1-a1-F	CCGGCTCCTGCTCCTCTTA	qRT-PCR
mCol1-a1-R	CCATTGTGTATGCAGCTGACTTC	qRT-PCR
MH1-F	AGGGAGACCCAAGCTTGCCACCATGGATGCCTGTCTGAGCATTG	SMAD4 domain mapping
MH1-R	GATATCTGCAGAATTCTGATACAACTCGTTCGTAGTGATATG	
linker-F	AGGGAGACCCAAGCTTGCCACCATGCCTGGAATTGATCTCTC	
linker-R	GATATCTGCAGAATTCAGCAGGATGATTGGAAATGGGAGGC	
MH2-F	AGGGAGACCCAAGCTGCCACCATGCCTGAGTATTGGTGTTC	
MH2-R	GATATCTGCAGAATTCATGAAGTACTTCGTCTAGGAGCTGG	
GST-sirt7-F	CCGCGTCGACATGGCAGCCGGGGGTCTGAG	purification of GST-tagged SIRT7
GST-sirt7-R	ATAGTTTAGCGGCCGCATTACGTCACTTTCTTCCTTTTTG	

P32-smad4-F	CGCGGATCCATGGACAATATGTCTATTAC	purification of 6XHis-tagged SMAD4
P32-smad4-R	CCCAAGCTTGTCTAAAGGTTGTGGGTCTG	
Delta-N-F	ATTCATCGATAGATCCGGGAGCTGGCCAGCGCCGT	SIRT7 domain mapping
Delta-N-F	ATGCCACCCGGGATCTTACGTCACTTTCTTCC	
CA-F	ATTCATCGATAGATCAGGCGCGGGAATCAGCACGG	
CA-R	ATGCCACCCGGGATCTTAGGGGATCTCCAAGCCCAG	
Delta-C-F	ATTCATCGATAGATCATGGCAGCCGGGGGTCTGAG	
Delta-C-F	ATGCCACCCGGGATCTTAGGGGATCTCCAAGCCC	
SMAD1-F	AGGGAGACCCAAGCTGCCACCATGAATGTGACAAGTTTATTTTC	Clone SMAD1
SMAD1-R	GATATCTGCAGAATTAGATACAGATGAAATAGGATTATGA	
SMAD2-F	AGGGAGACCCAAGCTGCCACCATGTCGTCCATCTTGCCATT	Clone SMAD2
SMAD2-R	GATATCTGCAGAATTTGACATGCTTGAGCAACGCA	
SMAD3-F	AGGGAGACCCAAGCTGCCACCATGTCGTCCATCCTGCCTTT	Clone SMAD3
SMAD3-R	GATATCTGCAGAATTAGACACACTGGAACAGCGGA	

KR385-F	GGTTGCACATAGGCAGAGGTGTGCAGTTGGAATG	
KR385-R	CATTCCAACTGCACACCTCTGCCTATGTGCAACC	
KR428-F	CTGGAGATGCTGTTCATAGGATCTACCCAAGTGC	
KR428-R	GCACTTGGGTAGATCCTATGAACAGCATCTCCAG	
KR507-F	CTCAGGATGAGTTTTGTGAGAGGCTGGGGACCGG	
KR507-R	CCGGTCCCCAGCCTCTCACAAAACTCATCCTGAG	
KR519-F	CCCAAGACAGAGCATCAGAGAAACACCTTGCTGG	SMAD4 mutation
KR519-R	CCAGCAAGGTGTTTCTCTGATGCTCTGTCTTGGG	
KQ428-F	CTGGAGATGCTGTTCATCAGATCTACCCAAGTGC	
KQ428-R	GCACTTGGGTAGATCTGATGAACAGCATCTCCAG	
KQ519-F	CCCAAGACAGAGCATCCAAGAAACACCTTGCTGG	
KQ519-R	CCAGCAAGGTGTTTCTTGGATGCTCTGTCTTGGG	
PyMT-F	GGAAGCAAGTACTTCACAAGGG	Genome typing
PyMT-R	GGAAAGTCACTAGGAGCAGGG	Genome typing
mSirt7-teton-F	TTAGTGAACCGTCAGATCGC	Genome typing
mSirt7-teton-R	CTCCGGGTCATCACACACCT	Genome typing

Supplementary Table 2. Sequences of primers, siRNAs and shRNAs used in this study.

Supplementary references

1 Gyorffy, B. *et al.* An online survival analysis tool to rapidly assess the effect of 22,277 genes on breast cancer prognosis using microarray data of 1,809 patients. *Breast cancer research and treatment* **123**, 725-731, doi:10.1007/s10549-009-0674-9 (2010).

2 Minn, A. J. *et al.* Lung metastasis genes couple breast tumor size and metastatic spread. *Proc Natl Acad Sci U S A* **104**, 6740-6745, doi:10.1073/pnas.0701138104 (2007).

3 Ciriello, G. *et al.* Comprehensive Molecular Portraits of Invasive Lobular Breast Cancer. *Cell* **163**, 506-519, doi:10.1016/j.cell.2015.09.033 (2015).

4 Hoeflich, K. P. *et al.* In vivo antitumor activity of MEK and phosphatidylinositol 3-kinase inhibitors in basal-like breast cancer models. *Clin Cancer Res* **15**, 4649-4664, doi:10.1158/1078-0432.CCR-09-0317
1078-0432.CCR-09-0317 [pii] (2009).

5 Chakrabarti, R. *et al.* Elf5 inhibits the epithelial-mesenchymal transition in mammary gland development and breast cancer metastasis by transcriptionally repressing Snail2. *Nat Cell Biol* **14**, 1212-1222, doi:10.1038/ncb2607
ncb2607 [pii] (2012).

6 Dupont, S. *et al.* FAM/USP9x, a deubiquitinating enzyme essential for TGFbeta signaling, controls Smad4 monoubiquitination. *Cell* **136**, 123-135, doi:10.1016/j.cell.2008.10.051 (2009).
